# The influence of terpenes on the release of volatile organic compounds and active ingredients to cannabis vaping aerosols†

**DOI:** 10.1039/d1ra00934f

**Published:** 2021-03-23

**Authors:** Jiries Meehan-Atrash, Wentai Luo, Kevin J. McWhirter, David G. Dennis, David Sarlah, Robert P. Jensen, Isaac Afreh, Jia Jiang, Kelley C. Barsanti, Alisha Ortiz, Robert M. Strongin

**Affiliations:** Department of Chemistry, Portland State University Portland Oregon 97207-0751 USA strongin@pdx.edu; Department of Civil and Environmental Engineering, Portland State University Portland Oregon 97207-0751 USA; Roger Adams Laboratory, Department of Chemistry, University of Illinois Urbana Illinois 61801 USA; Florascience Inc. Milwaukie OR 97222 USA; Chemical and Environmental Engineering, Center for Environmental Research and Technology, University of California-Riverside Riverside California 92521 USA

## Abstract

Dabbing and vaping cannabis extracts have gained large popularity in the United States as alternatives to cannabis smoking, but diversity in both available products and consumption habits make it difficult to assess consumer exposure to psychoactive ingredients and potentially harmful components. This work studies the how relative ratios of the two primary components of cannabis extracts, Δ^9^-tetrahydrocannabinol (THC) and terpenes, affect dosage of these and exposure to harmful or potentially harmful components (HPHCs). THC contains a monoterpene moiety and has been previously shown to emit similar volatile degradation products to terpenes when vaporized. Herein, the major thermal degradation mechanisms for THC and β-myrcene are elucidated *via* analysis of their aerosol gas phase products using automated thermal desorption-gas chromatography-mass spectrometry with the aid of isotopic labelling and chemical mechanism modelling. Four abundant products – isoprene, 2-methyl-2-butene, 3-methylcrotonaldehyde, and 3-methyl-1-butene – are shown to derive from a common radical intermediate for both THC and β-myrcene and these products comprise 18–30% of the aerosol gas phase. The relative levels of these four products are highly correlated with applied power to the e-cigarette, which indicates formation of these products is temperature dependent. Vaping THC–β-myrcene mixtures with increasing % mass of β-myrcene is correlated with less degradation of the starting material and a product distribution suggestive of a lower aerosolization temperature. By contrast, dabbing THC–β-myrcene mixtures with increasing % mass of β-myrcene is associated with higher levels of HPHCs, and isotopic labelling showed this is due to increased reactivity of β-myrcene relative to THC.

## Abstract 2

Cannabinoid and VOC emissions from vaping cannabis concentrates vary depending on terpene content, power level and consumption method.

## Graphical abstract

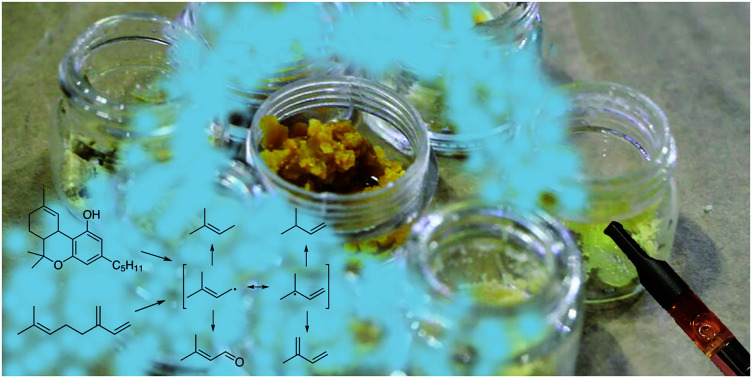

## Introduction

Humans have consumed cannabis for its psychoactive effect for as long as 2500 years^1^ and is the most consumed illicit substance worldwide.^2^ Smoking dried inflorescences in a pipe or cannabis cigarette remains the most popular mode of consumption,^3^ but novel inhalation methods have been recently developed^4^ with the purpose of avoiding toxic combustion by-products, and for more intense delivery of active ingredients and flavorings.^5^ Vaporizing or vaping cannabis has surged in popularity in the United States in all age groups,^6^ particularly among adolescents.^7^

The two primary methods for inhaling cannabis extracts are dabbing and vaping with cannabis e-cigarettes (CECs).^5,8^ Dabbing is performed by placing a small amount of cannabis extract onto a heated surface while the user takes a large inhalation of up to an entire inspiratory capacity (<3 L).^5,8^ CECs, commonly known as vape pens or oil pens, are compact e-cigarettes comprised of a single-use or refillable atomizer cartridge attached to variable or fixed-voltage batteries. The cartridge contains 0.3–1.0 g cannabis oil, a viscous substance that may contain up to 90% of the psychoactive Δ^9^-tetrahydrocannabinol (THC, mp = rt,^9^ bp = 416 °C (ref. 10)).^5^ Dabbing and CEC use have quickly surged in popularity, and one recent study showed 19.5% of past-month cannabis users reported CEC vaping, and 14.6% reported dabbing.^11^

**Fig. 1:**
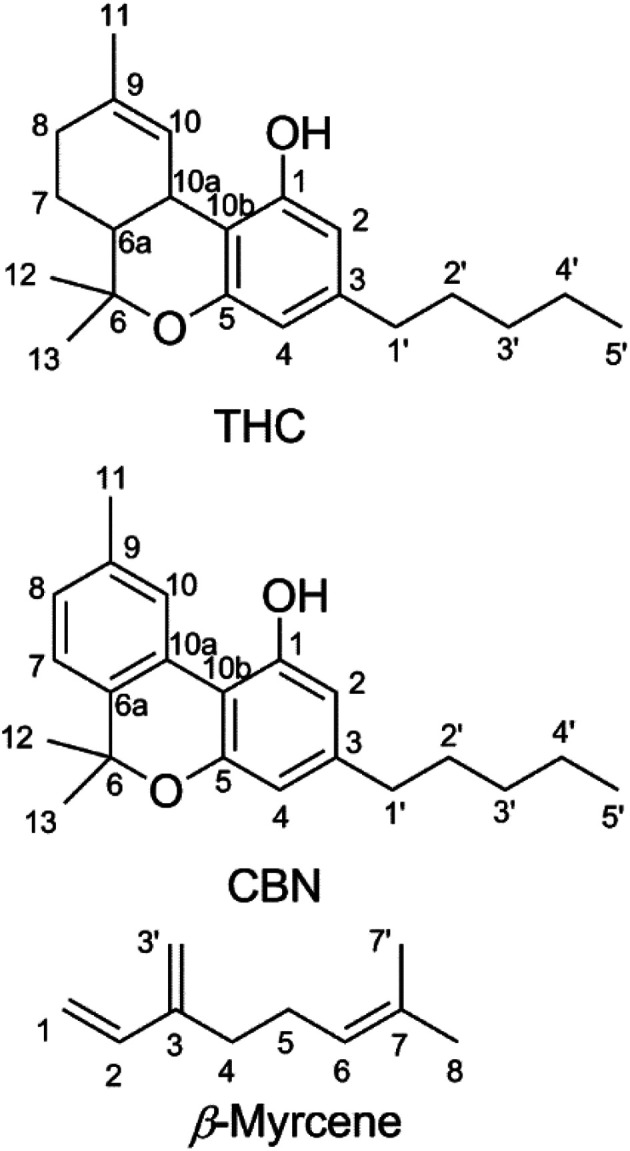
Chemical structures of Δ^9^-tetrahydrocannabinol (THC), cannabinol (CBN), and β-myrcene shown with carbons numbered.

Cannabinoids are expressed in *Cannabis sativa* as cannabinoid acids,^12^ with an aryl carboxy group at the 2-position of the phenol ring (Fig. 1).^13^ Δ^9^-Tetrahydrocannabinolic acid (THCA, mp = 70 °C (ref. 14)) decarboxylates readily to THC at temperatures seen in smoking^15,16^ and vaping.^17,18^ Butane extracts (butane hash oil, BHO) do not experience high temperatures during production,^19^ primarily contain cannabinoid acids^20^ and are solid. BHO is typically consumed by dabbing.^19^ Purification and decarboxylation using advanced techniques isolates neutral cannabinoids and cannabis terpenes which may be reconstituted and used in a CEC.^21^ In addition to adding flavor, terpene blends of cannabis-derived and synthetic or botanical terpenes^21^ also reduce the viscosity of THC which facilitates handling and administration.^22^ Other ingredients added as cutting agents^22–24^ are extremely controversial given the recent outbreak of e-cigarette or vaping product use-associated lung injury (EVALI), in which the viscosity modifier vitamin E acetate was implicated as a potential causative agent.^23,25,26^

Volatile Organic Compounds (VOCs) in cigarette smoke^27^ contribute 62% of the excess lifetime cancer risk associated with cigarette smoking.^28^ VOCs present in cannabis vaporizer aerosols are significantly different from those in tobacco and cannabis smoke. They consist largely of terpenes and terpene pyrolysis and oxidation products such as isoprene, methacrolein (MACR), methyl vinyl ketone (MVK), and 3-methylfuran, among others.^5,8^ Exposure to terpene oxidation products causes sensory irritation and airflow limitation in exposed mice,^29^ and gaseous products are indicated to be responsible for the majority of these symptoms.^30^ In humans, exposure to terpenes and terpene/isoprene oxidation products at concentrations typical of indoor air do not significantly cause airway inflammation or sensory irritation,^31^ but the impact of inhaling these products at concentrations orders of magnitude greater than in indoor air has not been thoroughly investigated.

Automated thermal desorption-gas chromatography-mass spectrometry (ATD-GC-MS) is a powerful analytical technique that allows the identification and quantification of gases at trace levels for applications such as the atmospheric analysis of anthropogenic VOCs,^32,33^ metabolomics,^34–36^ and materials analysis.^37,38^ In the e-cigarette aerosol analysis field, ATD-GC-MS has allowed the determination of gas/particle partitioning constants of e-cigarette ingredients^39^ including nicotine in heat-not-burn tobacco vaporizers,^40^ as well as the identification of myriad degradation products emitted by both nicotine and cannabis vaporizers.^5,8,41^

It was previously shown that the addition of ∼10% cannabis terpenes to THC was associated with an increase in the levels of all VOCs as compared to pure THC when these were subjected to dabbing.^5^ Herein, the degradation of a model cannabis terpene, β-myrcene, and THC are studied mechanistically, and a site-specifically isotopically-labelled β-myrcene is used to track this terpene's degradation during dabbing THC–β-myrcene mixtures. Given the popularity of CEC vaping, VOCs released by a popular CEC containing THC with variable terpene content are studied to investigate how added terpenes and applied power impact the nature and quantity of gas phase VOCs. Additionally, the impact of applied power on the release of HPHCs, terpenes, and THC per puff is investigated, providing insight into aerosolization efficiency and dosing of a popular type of cannabis vaporizer.

## Materials and methods

### Synthetic cannabis oil (SCO)

THC (Cayman Chemical, Ann Arbor, MI) was acquired as a 50 mg mL^−1^ solution in acetonitrile, which was concentrated *in vacuo*. Pure THC was assessed for purity by high performance liquid chromatography with UV-vis detection (HPLC-UV) and nuclear magnetic resonance spectroscopy (NMR). THC was used alone in vaping or dabbing experiments, or mixed with β-myrcene (Sigma Aldrich) or β-myrcene-*d*_6_ for studies using SCO. THC and β-myrcene mixtures were homogenized in scintillation vials using a rotary evaporator slowly spinning at atmospheric pressure with the vial partially submerged in a 50 °C water bath for 1–2 hours. THC content was assessed by HPLC-UV. See SI for β-myrcene-*d*_6_ synthetic methodology and spectral characterization.

### Dabbing and vaping

SCO containing β-myrcene-*d*_6_ and THC, pure β-myrcene-*d*_6_, and pure THC were subjected to dabbing as per a previously established dabbing protocol.^5^ A novel CEC vaping protocol is described herein for chemical analysis of the aerosol gas phase (GP) and quantification of THC in the particle phase. Aerosols were generated using a TH2 CCELL connected to an iStick PICO battery. Cambridge filter pads (CFPs) were used to collect and remove particulate matter (PM), and GP products were collected on sorbent tubes containing a mixture of Tenax TA and Carbograph 1 sorbent materials. Airflow was generated using a Cigarette Smoking Machine used to generate puffs replicating the e-cigarette puff profile defined by the Cooperation Center for Scientific Research Relative to Tobacco (CORESTA) (50 mL puff volume, 3 s puff duration).^42^ A mass flowmeter was used to monitor puff volume, and an average of 44 ± 3 mL volume and 0.87 ± 0.05 L min^−1^ flowrate were observed. The battery was manually activated which caused small variations in puff duration, but puff durations were not recorded. Variation in flowrate through the sorbent material caused differences in puff volume between samples, but no significant differences (*p* < 0.05) in flowrate or puff volume exist between any two sample sets. A single puff was collected per replicate to limit overloading the GC-MS. The vaporizer atomizer was weighed before and after each puff to obtain the mass consumed per puff (*m*_C_). See ESI† for further details.

### Aerosol gas phase analysis

Sorbent tubes were stored at −20 °C for not more than seven days before analysis. Sample tubes were desorbed using a TurboMatrix 650 automated thermal desorption unit, and were amended with internal standards prior to desorption. Following desorption, samples were trapped, desorbed and transferred to an Agilent 7890A gas chromatograph for separation, interfaced with an Agilent 5975C mass spectrometer (MS) for detection. See SI for further ATD-GC-MS details.

### THC transfer analysis

THC transfer per puff (THC_T_) was determined for CEC vaping experiments only. Aerosol PM analysis is sufficient for assessing THC_T_, as its low vapor pressure (2.6 × 10^−5^ Pa)^10^ affords it a high theoretically-calculated gas/particle partitioning constant (*K*_p_ = 0.31, calculated using Pankow [2001]^43^), with 100.00% partitioned to the aerosol PM. CFPs were extracted in 1 : 1 methanol : acetonitrile, added with an internal standard (olivetol), and analyzed for THC content by HPLC-UV on a six-point internal standard calibration curve. See ESI† for further details.

### Data analysis and statistics

Semi-quantitative cannabinoid and terpene dabbing experiments were performed in duplicate, and quantitative CEC vaping experiments were carried out using 3–6 replicates. For semi-quantitative ATD-GC-MS studies, single air blanks were collected and compounds present in the air were manually removed from sample data sets. For CEC vaping experiments, air blanks were collected in triplicate, and VOCs present in the air were quantified per volume unit of air, and the air-contribution of VOCs was accounted for. Quantification of GP analytes by ATD-GC-MS was performed by comparing their total ion chromatogram integrations to that of an internal standard (fluorobenzene or 1,2-dichlorobenzene-*d*_4_), assuming a 1 : 1 response factor. To provide higher accuracy for HPHCs with toxicological significance, their response factors relative to internal standard were determined by estimating their ionization cross section. Outliers were removed when appropriate using a Grubb's test performed at the 95% confidence level. All values are presented as *x̄* ± 95% confidence interval, unless otherwise noted, and all significance tests were performed considering *p* < 0.05. See ESI† for further details.

## Results & discussion

### The thermal degradation of β-myrcene

**Fig. 2:**
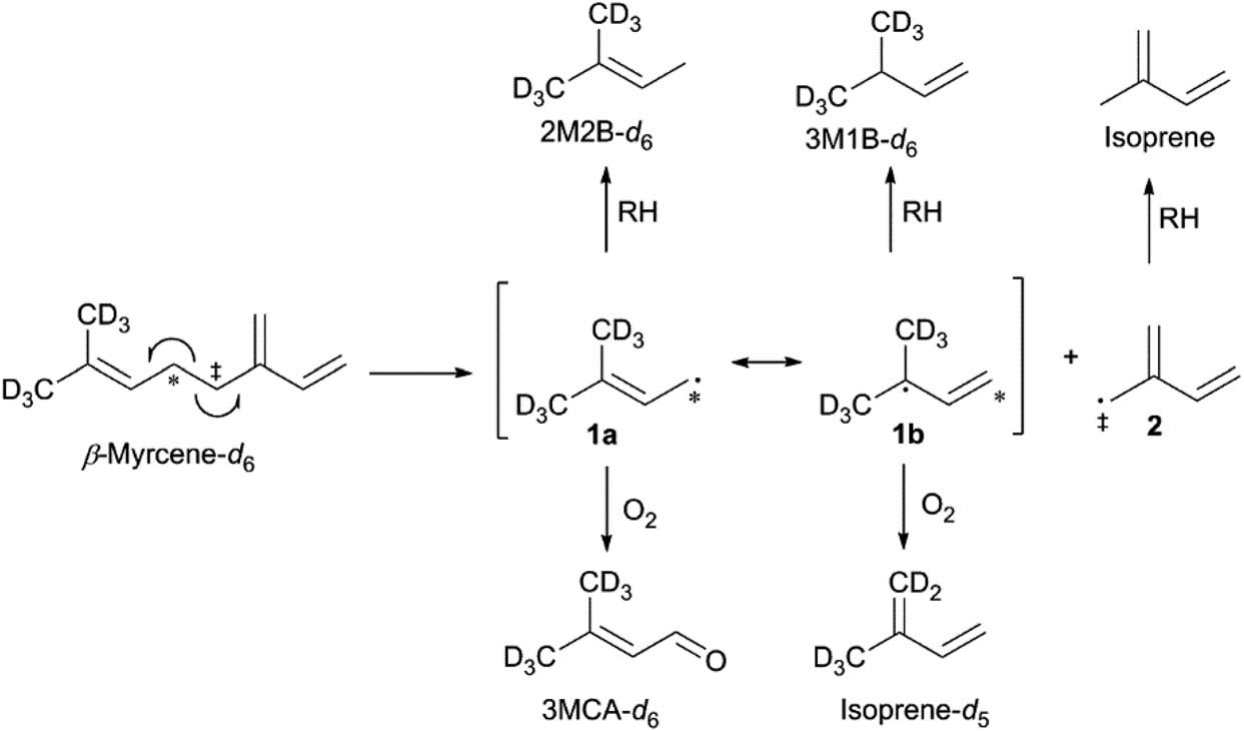
Proposed mechanism for the thermal degradation of β-myrcene-*d*_6_. The natural isotopologues of these reactions products compose ∼30% of the VOC_NT_ observed for β-myrcene.

The thermal degradation of β-myrcene, a ubiquitous and often dominant terpene present in many inhalable cannabis products, was characterized extensively herein to help reveal the influence of terpenes on dabbing and vaping using a CEC. A site-specifically isotopically-labelled β-myrcene, β-myrcene-*d*_6_ (Fig. 2) was subjected to dabbing, and isotopologues of known degradants were identified by examination of their mass spectra. A sample chromatogram is displayed in the ESI (Fig. S10†). The diversity of degradation products seen for β-myrcene dabbing suggest that many degradation pathways exist, but a mechanism can be ascribed to account for ∼30% of the formed VOCs, including the most abundant product, isoprene (Fig. 2). After homolytic cleavage between carbons 4 and 5,^44^ radicals 1 and 2 are formed. Resonance structure 1a undergoes oxidation to form 3-methylcrotonaldehyde-*d*_6_ (3MCA-*d*_6_), or is reduced by an alkyl R–H to form 2-methyl-2-butene-*d*_6_ (2M2B-*d*_6_). The tertiary radical 1b oxidizes to the isoprene deuterium isotopologue isoprene-*d*_5_, or undergoes reduction to 3-methyl-1-butene-*d*_6_ (3M1B-*d*_6_). Radical 2 undergoes reduction to isoprene, but no oxidation products of this radical are observed.

MACR and MVK, two abundant and toxicologically-concerning VOCs observed in all terpene and cannabinoid vaping experiments, are known isoprene oxidation products.^45,46^ During atmospheric oxidation of isoprene, the formation of MVK is more favorable than MACR due to its more stable reactive intermediates.^45,46^ For terpene and cannabinoid vaping experiments, a MACR : MVK ratio of ∼10 is typically observed,^5,8^ contrary to what would be expected.^45,46^ Two gas phase chemical mechanism generators and box models, SAPRC and GECKO-A, were used to derive chemical mechanisms for β-myrcene oxidation under vaping conditions; SAPRC was also used to predict levels of product formation in the vapor stream immediately following the heat source (simulation conditions: 300 ppm gaseous β-myrcene, 643 K). The chemical mechanism derived using GECKO-A was consistent with the experimentally derived mechanism supported by the deuterium incorporation in the isotopologues of MACR and MVK that were observed (MACR-*d*_5_ and MVK-*d*_3_, Fig. S8 and S9†). Importantly, SAPRC predicted an elevated MACR : MVK ratio that generally increased as a function of temperature and was ∼10 at 643 K. See ESI† for details regarding chemical mechanism modelling.

### Thermal degradation of Δ^9^-tetrahydrocannabinol

The thermal degradation of cannabinoids has been previously investigated from a chemical perspective with the focus on identifying novel, high molecular weight products that may have mutagenic or carcinogenic potential.^47^ Many of the chemical transformations observed involve the *p*-menthyl ring on THC and cannabidiol (CBD), and CBD pyrolysis products such as 2-methyl-5-pentylresorcinol and 5-pentylresorcinol indicate this terpenoid moiety may be lost entirely.^47–50^ GP degradants emitted by pure THC subjected to dabbing were previously reported by us, and as with the case for CBD, the *p*-menthyl moiety was hypothesized to be particularly labile given the high levels of isoprene, MACR, and other known terpene- and isoprene-derived degradants.^5^

Given the known topography associated with CEC vaping, THC degradation was investigated using this type of device to provide a per-puff-based quantitation of the VOCs released to the aerosol GP. Pure THC was introduced in a CCELL TH2 atomizer and the aerosol GPs from single puffs at 10 W using the CORESTA puffing topography for e-cigarettes were collected (in triplicate) and characterized by ATD-GC-MS. The resultant chromatograms display particularly elevated levels of isoprene, substituted C6–C10 dienes, and aromatics such as toluene and xylenes, with a total of 6.3 ± 0.4 μg of total VOCs (VOC_T_) in the aerosol GP quantified by non-target analysis. THC was also subjected to dabbing for qualitative analysis of its product distribution. See ESI† for a sample chromatogram, a full list of products tentatively identified.

In order to determine the origin of these degradation products, cannabinol (CBN, Fig. 1), was subjected to identical vaping conditions as THC. CBN is a THC oxidation product that forms during storage and processing.^51^ CBN shares identical structural features with THC except for the aromatic thymyl ring, and CBN has only limited psychoactivity when compared with THC.^52^ CBN vaporized in a CEC shows a starkly different aerosol GP that consists almost entirely of 1-butene, 1-propene, 1-pentene, butanal, propanal, and pentanal. C–C bond scission on the alkyl chain releases 1° alkyl radicals that form peroxy radicals after O_2_ addition, which subsequently undergo intramolecular rearrangement to hydroperoxy radicals that decompose to an alkene, or may undergo direct beta scission to an aldehyde. The quantity of VOCs released by CBN (0.6 ± 0.3 μg) is ∼10-fold lower than those released by THC vaporized under identical conditions.

**Fig. 3:**
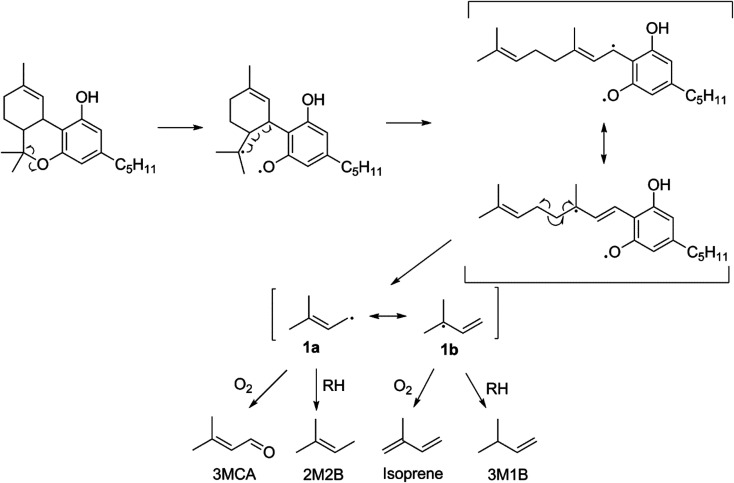
The proposed reaction scheme for a major thermal degradation pathway of THC which accounts for 22 ± 6% of VOC_T_ when THC is vaporized alone in a CEC at 10 W, and 18 ± 4% of VOC_T_ when THC is vaporized alone by dabbing at 370 °C.

The lack of isoprene and terpene-related degradation products in CBN's VOC profile is strong evidence that THC's *p*-menthyl ring accounts for the majority of THC's thermal degradation products. Moreover, the starkly increased quantity of VOCs (significant at *p* < 0.05) suggest this is a particularly labile structure. Fig. 3 is proposed pathway of THC decomposition accounting for 23 ± 6% of its VOC_T_ for vaping THC in a CEC. The initial bond scission between carbon 6 and O is likely the most thermodynamically favorable to occur in THC given the stability of the two resultant radicals (3° and phenoxyl). Subsequent beta scission opens the *p*-menthyl ring resulting in a cannabigerol-like diradical with a linear terpene moiety that readily decomposes to release the same radical formed during β-myrcene thermal degradation (1), and consequently, four of the same products are released: 3MCA, 2M2B, isoprene, and 3M1B. THC subjected to dabbing releases elevated levels of oxidation products, with 30 ± 10% (*n* = 2) carbonyls relative to all other GP products tentatively identified, which is significantly higher than THC vaporized in a CEC with 2.1 ± 0.9% (*n* = 4) carbonyls.

### Increased terpene content leads to elevated release of degradation products for dabbing

Many different types of dabbing apparatuses exist, but even for two consumers using the same device, the process by which they heat the nail, administer the dab, and take the inhalation may vary greatly. The two primary generalities that can be extrapolated are: the use of a nail, and a high inhalation volume. The experiments herein use an electrically heated titanium nail that is directly connected to CFP holder *via* a small glass adapter. Air flow generated by a laboratory vacuum pump is adjusted with a needle valve and monitored with a mass flow meter to generate enough flow (1–2 L min^−1^) so that the aerosol stream is pulled through the nail.

**Fig. 4:**
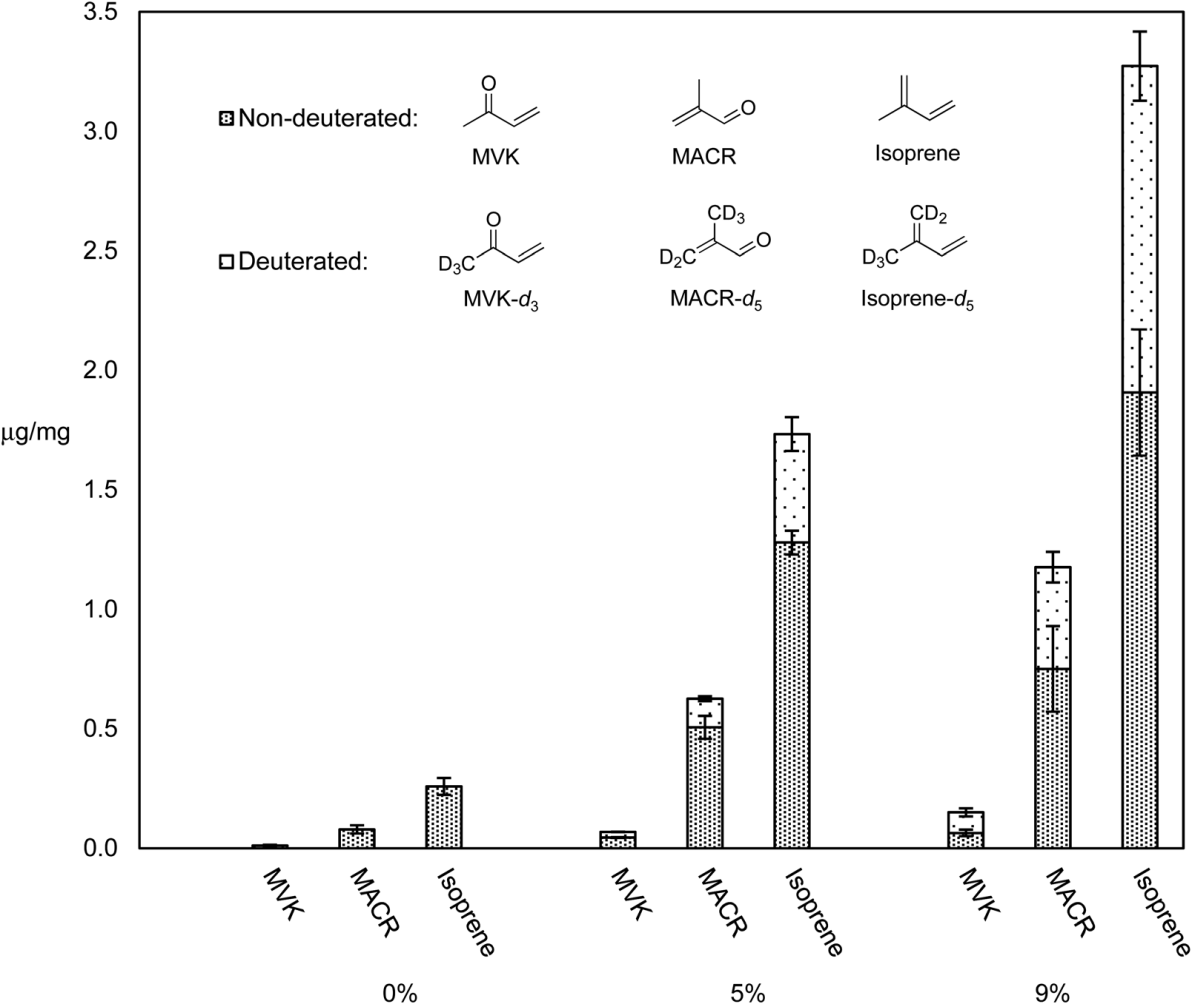
Comparative levels of major degradation products and their deuterated isotopologues encountered in the aerosol GP from dabbing pure THC (0% β-myrcene-*d*_6_), THC with 5% β-myrcene-*d*_6_, and THC with 9% β-myrcene-*d*_6_. Error bars are SEM.

We previously reported levels of HPHCs and all VOCs for dabbing a synthetic cannabis extract containing ∼10% of a cannabis terpenes mixture in THC, and showed that this mixture releases higher levels of all VOCs as compared to pure THC, and higher levels of selected toxicants compared to vaping a THC – terpene mix.^5^ It was hypothesized that terpenes may be more thermally labile than THC, and thus responsible for the increased quantity of degradation products. In order to test this, THC–β-myrcene mixtures were subjected to dabbing at 370 °C (a typical dabbing temperature^5^) using a previously reported dabbing method,^5^ and the levels of known degradants and their D-isotopologues were compared. Fig. 4 displays the levels of select degradants and their D-isotopologues as μg mg^−1^ of PM collected on CFPs for pure THC, THC with 5% β-myrcene-*d*_6_, and THC with 9% β-myrcene-*d*_6_.

Aerosol levels of major HPHCs known to exist when vaping cannabis oil components^5,8^ (isoprene, MACR, and MVK) increased with increasing % mass of β-myrcene-*d*_6_, and the elevated levels of their isotopologues that are known to derive from β-myrcene-*d*_6_ suggest this terpene was responsible for disproportionately more HPHCs compared to THC. Accounting for the isoprene–isoprene-*d*_5_ ratio of 0.45 ± 0.02 observed when pure β-myrcene-*d*_6_ is subjected to dabbing, in the THC–β-myrcene mixture containing 5% β-myrcene-*d*_6_, the terpene affords a 0.75% yield of isoprene, while THC produces only 0.15%. For the THC – β-myrcene mix containing 9% β-myrcene-*d*_6_, the terpene results in a 1.9% yield of isoprene, and THC a yield of 0.3%.

The higher yield of isoprene from β-myrcene may be explained *via* a combination of several factors. Isoprene has a more direct route to formation from β-myrcene than from THC, requiring less energy to generate this product. Additionally, β-myrcene partitions mostly to the aerosol GP, facilitating these reactions that are known to occur in this state.^45,46,53,54^ THC only has an appreciable distribution to GP at elevated temperatures directly surrounding the nail, but quickly condenses to PM, allowing less time for GP reactions to occur. β-Myrcene's smaller size and many fewer degrees of freedom than THC affords it a smaller molar heat capacity than THC, increasing the likelihood of bond homolysis with applied heat.

### Increased terpene content in cannabis oil decreases degradation and increases transfer of starting materials for cannabis e-cigarette vaping

VOCs released from vaping SCO in a CEC using THC and a commercially-available terpene mixture have been previously reported by us.^5^ Unlike the case with dabbing, this method's similarity to traditional nicotine e-cigarettes permits the usage of a standardized vaping topography (CORESTA^42^) in the experiments, and it is possible to extract quantitative data related to starting material transfer (THC and β-myrcene), the quantity of SCO consumed, and VOC emissions on a per-puff basis. As with the case with the above dabbing experiments, these experiments used β-myrcene as a model terpene to test how this cannabis oil component impacts aerosolization during vaping.

Pure THC, THC with 7.2% β-myrcene, and 14% β-myrcene were added to CCELL TH2 atomizers and vaporized at 10 W. Mass of SCO consumed (*m*_C_, Table 1) did not significantly change as β-myrcene % mass increased from 0% (pure THC) to 7.2%, and decreased non-significantly as % mass increased to 14%. THC_T_ increased significantly in a linear fashion (*R*^2^ = 0.99) with increasing β-myrcene % mass. THC yield (THC_Y_) increased significantly in a linear fashion (*R*^2^ = 0.98) upon increasing the β-myrcene % mass. β-Myrcene transfer (β-myrcene_T_) expectedly doubled as the % mass β-myrcene doubled from 7.2% to 14%, but the yield of β-myrcene (β-Myrcene_Y_) did not significantly change.

**Table 1:** CEC vaping experiments in which both terpene content and power level were studied to probe their effect on yields of active ingredients and degradation products. For the experiments wherein % mass β-myrcene was the variable, power level was kept at a constant 10 W. For the experiments wherein power level was varied, % mass β-myrcene in CVL was 14%

	% β-Myrcene in THC			Power		
	0%	7%	14%	8 W	10 W	12 W
*n*	4	6	5	3	5	3
*m*_C_ (mg)	5 ± 3	5 ± 4	7 ± 3	4 ± 1	7 ± 3	7 ± 2
THC_T_ (mg)	1.6 ± 0.6	3 ± 2	4 ± 1	2.9 ± 0.2	5 ± 1	5 ± 1
THC_Y_ (%)	4 × 10^1^ ± 2 × 10^1^	5 × 10^1^ ± 2 × 10^1^	8 × 10^1^ ± 1 × 10^1^	9 × 10^1^ ± 3 × 10^1^	8 × 10^1^ ± 1 × 10^1^	8 × 10^1^ ± 1 × 10^1^
β-Myrcene_T_ (μg)	0 ± 0	8 ± 5	17 ± 6	18 ± 4	17 ± 8	12 ± 3
β-Myrcene_Y_ (%)	NA	2.2 ± 0.6	1.8 ± 0.9	3.3 ± 0.4	1.8 ± 0.9	1.4 ± 0.4
psi-Limonene_D_ (μg)	0 ± 0	3 ± 3	9 ± 3	9 ± 2	9 ± 4	6 ± 2
VOC_NT_ (μg)	6.3 ± 0.4	9 ± 4	5 ± 1	3 ± 1	5 ± 1	9 ± 2
Isoprene (μg)	1.35 ± 0.04	1.5 ± 0.5	0.5 ± 0.2	0.07 ± 0.02	0.5 ± 0.2	1.5 ± 0.1
Isoprene epoxide (ng)	7 ± 4	5 ± 3	3 ± 1	0.59 ± 0.01	3 ± 1	4 ± 3
1,3-BD (ng)	12 ± 8	13 ± 9	3 ± 1	3 ± 1	3 ± 2	6 ± 8
MACR (ng)	41 ± 3	4 × 10^1^ ± 2 × 10^1^	16 ± 5	5 ± 2	16 ± 8	31 ± 9
MVK (ng)	39 ± 3	5 × 10^1^ ± 2 × 10^1^	22 ± 4	5 ± 7	22 ± 6	4 × 10^1^ ± 2 × 10^1^
Butanal (ng)	11 ± 3	7 ± 2	5.8 ± 0.8	0.8 ± 0.2	6 ± 1	4 ± 2
Benzene (ng)	10 ± 4	3 × 10^1^ ± 4 × 10^1^	2 ± 2	0 ± 0	2 ± 3	4 ± 3
Toluene (ng)	1 × 10^2^ ± 2 × 10^1^	2 × 10^2^ ± 2 × 10^2^	2 × 10^1^ ± 1 × 10^1^	10 ± 7	3 × 10^1^ ± 1 × 10^1^	8 × 10^1^ ± 5 × 10^1^
Xylenes (ng)	2.4 × 10^2^ ± 3 × 10^1^	4 × 10^2^ ± 4 × 10^2^	2 × 10^1^ ± 2 × 10^1^	2 × 10^1^ ± 2 × 10^1^	2 × 10^1^ ± 3 × 10^1^	1 × 10^2^ ± 1 × 10^2^

Some HPHCs previously identified in the cannabis vaporizer aerosol GP that have a calculated inhalation unit risk or reference exposure level values with regard to their cancer or non-cancer chronic exposure risk were measured and are displayed in Table 1.^5^ Isoprene epoxide was identified in all ATD-GC-MS chromatograms, and quantitative data for this compound was also included in Table 1 as this molecule is known to mediate the mutagenic effect of isoprene.^55^ Overall, the highest β-myrcene % mass tested, 14%, resulted in the lowest overall delivery of HPHCs. Pure THC and the SCO with 7.2% β-myrcene release similar levels of all HPHCs.

These results suggest THC and terpene transfer occur with less degradation as terpene % mass increases, and that the vaporizer operates with higher overall efficiency at the highest terpene % mass tested, 14%. The lower boiling point of β-myrcene (167 °C (ref. 56)) compared to THC (417 °C (ref. 10)) may translate to a reduced boiling point of the mixture, depressing the aerosolization temperature. β-Myrcene's enthalpy of vaporization may further depress reaction temperature. In addition to these effects, the observably lower viscosity of 14% β-myrcene likely facilitates wicking and improves atomizer efficiency.

### Applied electrical power increases degradation products and decreases transfer of starting materials for cannabis e-cigarette vaping

Herein we report the influence of power level applied to the CEC atomizer on the release of active ingredients and VOCs from an idealized cannabis e-cigarette that contains THC with 14 % mass β-myrcene, a composition seen in many available products.^21^ Two power levels above and below an acceptable and recommended power level for CCELL atomizers (10 W (ref. 57 and 58)) were used in this investigation: 8, 10, and 12 W. The relationship between power level at the atomizer and active ingredient transfer for vaporized THC with 14 % mass β-myrcene in a CEC displayed both linear and non-linear correlations (Table 1). THC_T_ and *m*_C_ both increased significantly from 8–10 W, but did not significantly change from 10–12 W. Correspondingly, THC_Y_ decreased significantly from 8–10 W, but did not significantly change from 10–12 W.

The observation of pseudolimonene (psi-limonene, Fig. S12†) in the ATD-GC-MS chromatogram of the aerosol was unexpected, but this product has been reported as a byproduct of β-myrcene synthesis *via* pyrolysis of β-pinene.^59^ psi-Limonene occurred at a near-uniform 1 : 2 ratio (β-myrcene : psi-limonene = 2.04 ± 0.04) when vaping the 14% β-myrcene in THC. Levels of β-myrcene_T_ and psi-limonene_T_ did not significantly change from 8–10 W but decreased significantly as power increased from 10–12 W. Correspondingly, β-myrcene_Y_ significantly decreased from 8–10 W and 10–12 W in a linear fashion (*R*^2^ = 0.92). VOC_NT_ increased significantly from 8–10 W and 10–12 W in a linear fashion (*R*^2^ = 0.95).

With regards to the release of HPHCs to the aerosol GP from vaping synthetic SCO, power level increased the amount of HPHC delivered per puff (Table 1). Linear correlations (all *R*^2^ > 0.9) are observed for isoprene, MACR, MVK, benzene, toluene, and isoprene epoxide. Butanal, xylenes, and butadiene displayed non-linearities that likely stemmed from integration error, which may be remedied by external calibration for more accurate data if necessary. Together these results indicate that this type of vaporizer should ideally be operated at the lowest power setting possible to avoid degradation of the starting material and production of HPHCs.

### Terpene and power levels influence the major degradation pathway of THC and β-myrcene during cannabis e-cigarette vaping

Reaction products that derive from the major degradation pathways of β-myrcene and THC show a dependence on both % mass β-myrcene and applied power suggesting that the 1a ↔ 1b equilibrium may be impacted by these factors. To assess relative levels of the oxidation and reduction products of this radical, integrations of the molecular ion for each species on the ATD-GC-MS chromatogram were obtained, and the relative levels of 1a to 1b products were calculated by summing the molecular ion or base peak integrations of 3MCA (*m*/*z* = 84 amu) and 2M2B (*m*/*z* = 70 amu) for 1a, and those of isoprene (*m*/*z* = 67 amu) and 3M1B (*m*/*z* = 70 amu) for 1b.

**Fig. 5:**
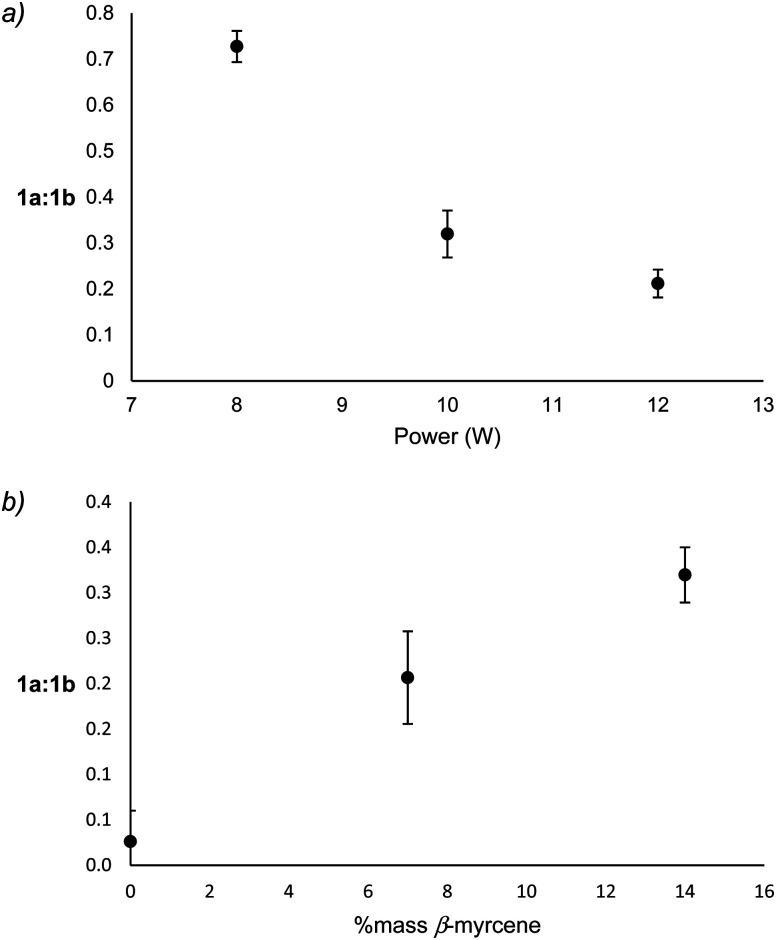
The relationship between applied power to 1a : 1b (a) and % mass β-myrcene to 1a : 1b (b). 1a : 1b is calculated as the quotient of the selected ion chromatogram integrations of the molecular ions for 1a products, 3MCA (*m*/*z* = 84 amu) and 2M2B (*m*/*z* = 70 amu), with 1b products, isoprene (*m*/*z* = 67 amu) and 3M1B (*m*/*z* = 70 amu).

Though it is not possible to measure the exact temperature experienced at the atomizer, it may be assumed that power level is directly related to aerosolization temperature. With increasing power, 1a-derived products decrease relative to 1b-derived products, a correlation that is largely governed by an increase of isoprene relative to 3MCA (see ESI†). The formation of 3MCA begins with O_2_ addition to C˙ on 1a to form a COO˙ species, which decomposes *via* C–H beta scission to yield 3MCA and a hydroxyl radical.^60^ Isoprene similarly begins with O_2_ addition at C˙ on 1b to form an RO_2_ radical which can directly release a hydroperoxyl radical and isoprene.^61^ At lower temperatures, the reversible addition of O_2_ onto C˙ faces a high barrier in the back reaction for 1a as this releases a primary radical, leading to an abundance of 3MCA as an end product. It is known that at higher temperatures, the barrier for O_2_ addition on any C˙ becomes nearly nonexistent.^61^ This favors oxidation *via* the more stable resonance contributor, 1b, at higher temperatures. 3MCA may be considered a kinetic product favored at low temperatures, and isoprene a thermodynamic product favored at higher temperatures. Significant decreases of the ratio of 1a : 1b products with increasing power support this hypothesis (Fig. 5a). Significant increases in 1a : 1b products with increasing % mass β-myrcene (Fig. 5b) suggest that vaping conditions with higher % mass β-myrcene occur at lower temperatures, which is supported by the observation of lower levels of degradation products and higher yield of starting materials under these conditions.

## Conclusions

Terpenes are shown to have a significant impact on aerosolization in both dabbing and CEC vaping. Curiously, opposite effects are observed for these two cannabis inhalation methods: higher levels of β-myrcene produces elevated levels of HPHCs during dabbing, but higher β-myrcene levels in SCO leads to lesser degradation and lower HPHC release for CEC vaping. For dabbing, this result is described using isotopic labelling, and it is shown that β-myrcene is more thermally labile than THC. The surface upon which aerosolization occurs is pre-heated to a desired temperature prior to administration of the material, and therefore all its components are subjected to the same temperature. Isotope labelling experiments indicate that β-myrcene has a 5–6 fold higher % yield of isoprene than THC. More facile routes to gaseous degradants, higher partitioning to the GP, and lower molar heat capacity are all factors that may explain the more extensive β-myrcene degradation compared to THC. Analogous findings consistent with this trend are likely for other terpenes with similar vapor pressures and molecular masses. Cannabis extracts used for dabbing typically contain cannabinoid acids, but these were not studied in this work given their lack of commercial availability for federally-funded academic research institutions in the United States of America as of this writing.

Conversely, higher β-myrcene % mass is associated with a decrease in the levels of all HPHCs and lesser overall degradation for CEC vaping. Less degradation and higher overall operating efficiency was observed when vaping SCO with higher % mass β-myrcene, likely a consequence of decreases in boiling point and viscosity. Depression of the boiling point would correspondingly depress aerosolization temperature in the atomizer and lead to lesser chemical degradation. Using the β-myrcene % mass that displays optimum performance, 14%, the influence of power level on VOC profile and THC content in the PM was examined. The increase in THC_T_ and decrease in THC_Y_ from 8–10 W, which plateaus from 10–12 W suggests that even at 10 W degradation of the starting material becomes significant.

In the United States state-level legal recreational cannabis market, reconstituted cannabis oils containing cannabinoids and terpenes are the norm for CECs,^21^ but vaporizers of black market origin are known to contain non-cannabis additives such as medium chain triglyceride oil, triethyl citrate, or phytol.^23^ The findings herein may not translate to cannabis vaporizer liquids containing these and other additives, though future work may investigate the impact of these on the release of VOCs and the delivery of THC and other aerosol components.

## Author contributions

JMA and RMS designed the experiments and wrote manuscript draft. JMA and AO performed vaping experiments, HPLC experiments, characterization, and analysis. WL and KJM designed and performed ATD-GC-MS experiments. DGD and DS designed and synthesize the β-myrcene-*d*_6_. RPJ synthesized CBN and aided in experimental design. IA, JJ, and KCB designed and performed the computational experiments. All authors approved the final version of the manuscript.

## Conflicts of interest

RPJ is a founder and Vice President of Florascience Inc., an Oregon hemp company. All other authors have no conflicts to declare.

## Supplementary Material

RA-011-D1RA00934F-s001
