# Supplementary material for: The influence of terpenes on the release of volatile organic compounds and active ingredients to cannabis vaping aerosols
Source: RSC Adv. 2021 Mar 23;11(19):11714–23. doi: 10.1039/d1ra00934f (PMC8695911; doi:10.1039/d1ra00934f)
Supplement: RA-011-D1RA00934F-s001 [file RA-011-D1RA00934F-s001.pdf]

Electronic Supplementary Material (ESI) for RSC Advances.
This journal is © The Royal Society of Chemistry 2021

Supporting Information:

The influence of terpene flavor additives on the release of volatile organic compounds and active ingredients to cannabis vaping aerosols

Jiries Meehan-Atrash,[†] Wentai Luo,[†,‡] Kevin J. McWhirter,[‡] David G. Dennis,[§] David Sarlah,[§] Robert P. Jensen,[||] Isaac Afreh,[#] Jia Jiang,[#] Kelley C. Barsanti,[#] Alisha Ortiz,[†] Robert M. Strongin.*[,†]

[†] Department of Chemistry, Portland State University, Portland, Oregon 97207-0751, USA

[‡] Department of Civil and Environmental Engineering, Portland State University, Portland, Oregon 97207-0751, USA

[§]Roger Adams Laboratory, Department of Chemistry, University of Illinois, Urbana, Illinois 61801, USA
[||]Floraworks Holdings Inc.

[#]Chemical and Environmental Engineering, Center for Environmental Research and Technology, University of California-Riverside, Riverside, California, 92521, USA

Table of Contents

Scheduled substance usage S3
Synthesis of β-myrcene-d_6 .... S3
Figure S1 .... S3
Synthetic cannabis oil .... S3 – S4
Cartridge vaping experiments .... S4 – S5
Figure S2 .... S4
THC delivery analysis .... S4 – S5
HPLC-UV methodology S5
ATD-GC-MS methodology S5
VOC quantification by ATD-GCMS .... S5 – S6
Computational modelling experiments S6
Figure S3 .... S6
Figure S4 .... S7
Figure S5 .... S7
Figure S6 .... S8
Figure S7 .... S8
Figure S8 .... S9
Figure S9 .... S9
Figure S10 .... S10
Figure S11 .... S10

Table S1 ....S10 – S12

Table S2 ....S12 – S14

Figure S12 ....S14

1a and 1b product distribution as a function of applied power.......S14 – S15

Figure S13 ....S14

Figure S14 ....S15

Figure S15 ....S15

Figure S16 ....S15

References S16

Scheduled substance usage. Research activities involved THC were performed in accordance with 21 C.F.R. §1301.18 and safely stored in accordance with §1301.75. THC was purchased from Cayman Chemical (Ann Arbor, MI) as a solution in acetonitrile at 50 mg/mL. The solvent was removed *in vacuo* before use in experiments. Cannabinol was graciously donated by Floraworks Holdings Inc.

Synthesis of *β*-myrcene-*d*$_6$. To a solution of hexadeutero isopropyl triphenylphoshine iodide salt (420 mg, 1.0 mmol, 1.1 eq) in THF (9 mL, 0.1 M) at 0 ^{0}C was added n-butyllithium (1.6 M, 620 µL, 1.0 mmol, 1.1 eq). This solution was allowed to stir at 0 ^{0}C for 30 min before a solution of 4-methylenehex-5-enal (100 mg, 0.90 mmol, 1.0 eq) in THF (0.50 mL) was added dropwise. The ice bath was removed and the reaction was permitted to stir at room temperature for 2 hours before being quenched with saturated aqueous ammonium chloride and extracted with pentane. The combined organic fractions were dried over anhydrous magnesium sulfate, concentrated under reduced pressure, and purified via flash chromatography (100% pentane) to provide the title compound in 54% yield in a 6:1 ratio with pentane. As expected, NMR analysis shows a spectrum identical to that of myrcene except for the absence of six proton signals associated with the geminal dimethyl olefin, and confirming the presence of 7-(methyl-*d*$_3$)-3-methyleneocta-1,6-diene-8,8,8-*d*$_3$ (*β*-myrcene-*d*$_6$). ^{1}H NMR (500 MHz, $CDCl_3$): δ 6.38 (dd, J = 17.6, 10.8 Hz, 1H), 5.25 (d, J = 17.6 Hz, 1H), 5.16 (t, J = 6.7 Hz, 1H), 5.03 (m, 3H), 2.20 (m, 4H).[1-4]

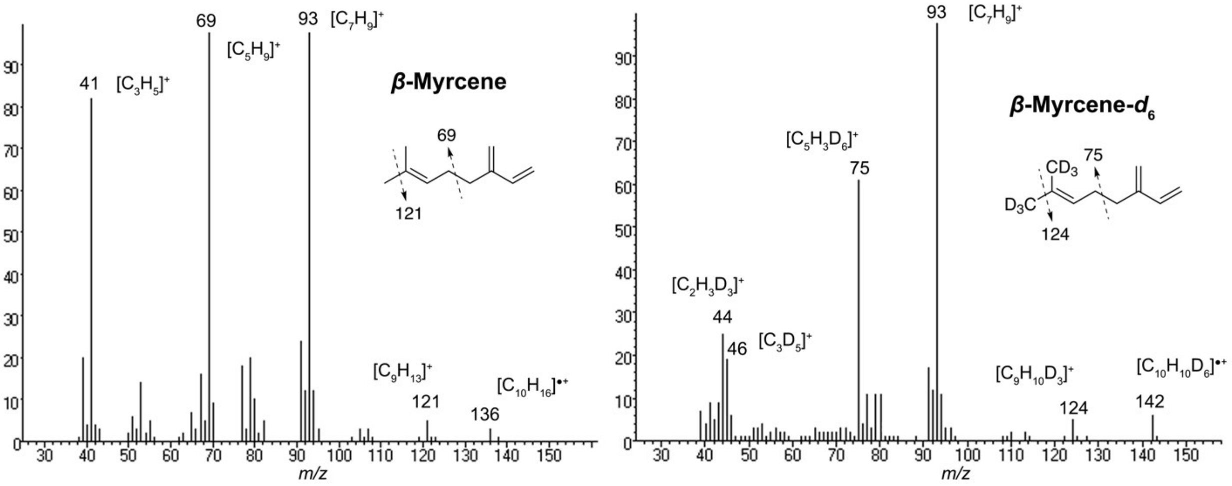

Figure S1. EIMS spectra of *β*-myrcene and *β*-myrcene-*d*$_6$

Synthetic cannabis oil. THC (Cayman Chemical, Ann Arbor, MI) was acquired as a 10 mg/mL solution in acetonitrile, which was concentrated *in vacuo*. Pure THC was assessed for purity by HPLC-UV and NMR. THC was used alone in vaping or dabbing experiments, or mixed with *β*-myrcene (Sigma Aldrich) or *β*-myrcene-*d*$_6$ for studies using synthetic cannabis oil. THC and *β*-myrcene mixtures were homogenized in scintillation vials using a rotary evaporator slowly spinning at atmospheric pressure with the vial partially submerged in a 50 ^{0}C water bath for 1 -2 hours. THC content was assessed by HPLC-UV on 5-point standard addition calibration curves by first creating analyte stock solutions. of the mixes at 1 -1.3 mg/mL in 1:1 CH_3CN:H_2O. 400 µL of 1.0 mg/mL (-)-Δ^9-THC in methanol certified reference material standard soln. (Cerilliant Corporation, Round Rock, TX) were added to a 2 mL vol. flask, and the methanol was

evaporated under a gentle stream of Ar, then brought up to volume in 1:1 $CH_3CN:H_2O$ for a final conc. of 200 μg/mL (THC spike soln.). 50 μL of analyte stock soln. and 100, 150, 200, 300, or 400 μL of THC spike soln. were added and to 2 mL. vol. flasks and brought up to volume in 1:1 $CH_3CN:H_2O$, and immediately analyzed by HPLC-UV monitoring at 254 nm.

Cartridge vaping experiments. Pure THC, THC with 7.2 % myrcene, THC with 14 % myrcene, and pure CBN were added to CCELL TH2 oil vape atomizer (CCELL) and warmed in a 40 ^{0}C oven for 3 -4 hours oven to allow the oil to saturate the internal wick, and then used the following day in vaping experiments. The atomizers were connected to an iStick PICO (eLeaf) battery that was set to the wattage required for each experiment. The aerosol collection apparatus (Figure S2) consisted of: the CEC atomizer/battery for aerosol generation, a 47 mm glass fiber filter pad (i.e. Cambridge filter pad [CFP], Healthcare) for aerosol particulate matter collection, a ¼" x 3.5" ATD sorbent tube containing 100 mg 35/60 mesh Tenax TA and 200 mg 60/80 mesh Carbograph 1 TD (Camsco Inc., Houston, TX), a 0 -10 L/min GFM Mass Flowmeter (Aalborg, Orangeburg, NY), and a Cigarette Smoking Machine CSM-STEP (CH Technologies).

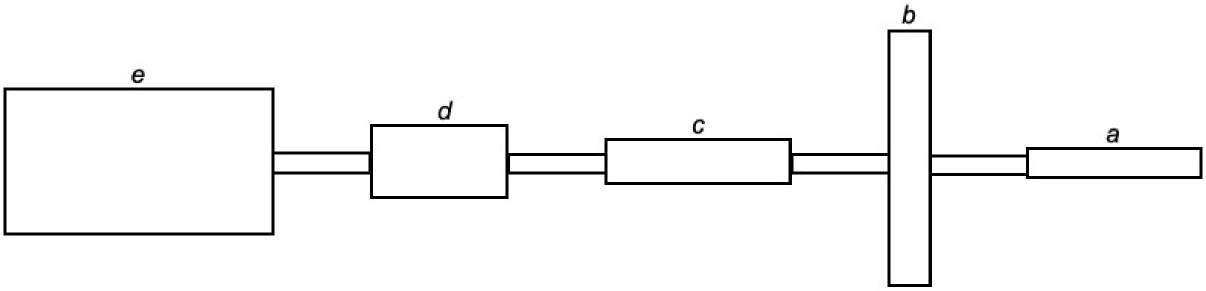

Figure S2. Aerosol collection apparatus for CEC vaping. *a:* CEC/battery; *b:* CFP holder; *c:* sorbent tube; *d:* mass flowmeter; *e:* CSM.

Given the variability of sorbent material packing in each ATD sorbent tube, each tube was calibrated on a 5-point calibration curve (CSM puff depth [V] vs. flowmeter flowrate [L/min]) in order to determine the puff depth setting on the CSM to match, as closely as possible, the CORESTA recommended setting for e-cigarette puffing: 50 mL puff volume in 3 s.[5] Knowledge of the exact puff volume facilitated air blank VOC correction. After calibration, VOC emissions from a single puff from the vaporizer were collected on the ATD sorbent tube, and the atomizer was massed before and after each puff. Air blanks were collected in triplicate in the exact same manner on the days experiments were performed and used to account for background levels of target VOCs in the samples. Benzene and toluene were the only target VOCs (Table 1) detectable. Air levels of benzene (4.3±0.2 ng/L) and toluene (2.0±0.4 ng/L) were taken as the mass of analyte collected on the sorbent tube vs. the total sampled air volume, including the calibration draws. Background contributions of benzene and toluene were subtracted from measured benzene and toluene levels in ATD sorbent tubes for vaping samples by accounting for the total sampled air volume for each (including calibration draws).

THC delivery analysis. Cambridge filter pads from CEC vaping experiments were extracted in 20 mL 1:1 $CH_3CN:H_2O$ added with 1 mL of an internal standard solution (5.574 mg/mL olivetol in 1:1 $CH_3CN:H_2O$). Olivetol was chosen as an internal standard due to its similar solubility to

THC, and its favorable retention time on the chromatogram relative to THC. Extraction solutions were stored at -20 °C for <2 days prior to analysis by HPLC-UV. THC concentration loss under these storage conditions was monitored, and concentration loss as monitored by HPLC-UV was only detectable after ~5 days. THC_D was quantified using a freshly-prepared six-point internal standard calibration curve with 0.0, 4.5, 9.1, 18.2, 36.4, and 59.1 µg/mL THC with 50.7 µg/mL olivetol in each.

HPLC-UV methodology. The following method was adapted from Protti *et al.* (2019).[6] A Waters 1525 Binary HPLC Pump with a Waters 2996 Photodiode Array Detector were used for the analysis. A 5 µL loop was loaded with 5x sample volume and copious wash solvent between injections to avoid contamination. Sample injection were separated over an Acclaim™ RSLC Polar Advantage II 3µm 120 Å 3.0x75 mm stationary phase. Mobile phase consisted of: solvent A, 0.1 % formic acid (Fisher Scientific) in HPLC-grade water (Honeywell, Morris Plains, NJ); solvent B 0.1 % formic acid (Fisher Scientific) in HPLC-grade acetonitrile (Honeywell, Morris Plains, NJ). The gradient separation was as follows: initially 50 % A, ramping down to 5 % A after 7 min., maintaining for 1 min., then ramping back to 50 % A for 1 min., with a re-equilibration time of 4 min. at 50 % A, for a total run time of 13 min. with combined flowrate of 0.3 mL/min. 3 -4 injections of a check standard (200 µg/mL THC) were performed prior to analysis to ensure retention time stability.

ATD-GC-MS methodology. Sorbent tubes were stored at -20 °C for not more than seven days before analysis. ATD sorbent tubes were thermally desorbed with a TurboMatrix 650 automated thermal desorber (ATD) unit. 20 ng fluorobenzene, 18.6 ng toluene-d_8, 21.7 ng 4-bromofluorobenzene, and 20.3 ng 1,2-dichlorobenzene-d_4 were added automatically to all ATD sorbent tubes prior to desorption as internal standards. The ATD unit thermally desorbed tubes for 8 min. at 285 °C with a He desorption flow of 40 mL/min and a split flow of 100 mL/min, and the desorption stream was trapped at -5 °C on an intermediate "Tenax trap." This intermediate trap was desorbed at 295 °C at a constant pressure of 35 psi on a split flow of 20 mL/min for 6 min. Through a 1m long and 0.25 mm i.d. deactivated, fused silica transfer line maintained at 235 °C, the sample stream was passed along to a 60 m, 0.25 mm i.d., and 1.4 µm film thickness Agilent (Santa Clara, CA) DB-VRX capillary GC column mounted in an Agilent 7890 A GC. The GC was interfaced with an Agilent 5975C MS in electron impact ionization at 70 eV in the positive ion mode, with an MS scan range of 34 -600 amu, and an electron multiplier voltage of 1725 V. GC oven temperature was held at 45 °C for 10 min, raised to 190 °C at 12 °C/min and held for 2 min, then raised to 240 °C at 6 °C/min and held for 5 min, then programmed down to 210 °C at 10 °C/min.

VOC quantification by ATD-GCMS. For all samples excluding those generated from the THC-*β*-myrcene-d_6 mixes, VOCs in the aerosol GP were quantified using the non-target analysis method from Meehan-Atrash *et al.* (2019).[7] Where selected HPHCs were quantified, an ionizaton cross section is calculated to provide a more accurate result. When total the yield of total VOCs (VOC_T) were calculated, the ionization cross section of all components of the chromatogram was assumed to be equal to that of a chosen internal standard, fluorobenzene.

In GP samples generated from THC-β-myrcene-d_6 mixes, the coeluting deuterated and non-deuterated compounds prevented these from being estimated using the above non-target analysis method, which requires integration on the total ion chromatogram. To overcome this, response factors for HPHCs of interest were determined from previously collected quantitative ATD-GC-MS chromatograms. The mass of each HPHC in the sample ($m_{\mathrm{HPHC,\ sample}}$, ng) per mg particulate matter collected (m_{PM}) was determined using equation 1:

$$m_{HPHC,\,sample}/m_{PM} = \frac{A_{HPHC}/A_{FB} \times RF_{FB}/RF_{HPHC} \times m_{FB} - m_{HPHC,\,blank}}{m_{PM}} \qquad \text{Eq. 1}$$

where A_{HPHC} is the area of HPHC's ion of interest in the selected ion chromatogram (SIC), A_{FB} is the m/z = 96 SIC area of the fluorobenzene internal standard, RF_{FB} fluorobenzene's response factor for m/z = 96 calculated from a blank run ($A_{m/z=96}/m_{\mathrm{FB}}$), RF_{HPHC} is the response factor of the HPHC's ion of interest calculated from an injection of pure standards, m_{FB} is the mass of fluorobenzene added (20 ng) to each sample, and $m_{\mathrm{HPHC,blank}}$ is the mass of HPHC present in the laboratory air blank. The response factor for a specific ion of interest of an HPHC was used for the equivalent ion in a deuterium isotopologue. For example, the RF for isoprene's m/z=67 amu ion was assumed to be equal to isoprene-d_5's m/z=71 amu ion, because these both occur after loss of a methyl hydrogen.

Chemical mechanism modeling. A gas-phase oxidation mechanism for β-myrcene was derived using the SAPRC8-9 mechanism generation system, MechGen10, and product formation was predicted using a SAPRC box model. MechGen uses experimentally derived rate constants and branching ratios if data are available and otherwise uses estimated rate constants and branching ratios based on group additivity and other estimation methods. MechGen has been used previously in the development of the SAPRC-18 mechanism11 and in development of a detailed SAPRC furans mechanism for atmospheric modeling.12 In this work, MechGen was used to derive a β-myrcene oxidation mechanism under vaping conditions (significantly higher VOC levels and temperature than atmospheric conditions); the MechGen-derived mechanism was then implemented into a SAPRC box model to simulate vaping of a β-myrcene (300 ppm) and THC (700 ppm) mixture at 643 K and 1 atm with 5 ppb of NO. The SAPRC simulation duration was 10 minutes with a time step of 0.1 min, and the OH level was controlled between 2×10-8 and 5×10-7 ppm throughout the simulations. The SAPRC modeling was used to investigate observed ratios of product formation as a function of temperature and NO level.

To further investigate product formation mechanisms, a second gas-phase chemical mechanism generator, GECKO-A, was used to derive a β-myrcene oxidation mechanism under vaping conditions. GECKO-A is a nearly explicit chemical mechanism generator that relies on experimental data, structure-activity relationships, and a predefined protocol to generate detailed oxidation reaction schemes for organic compounds under atmospheric conditions (Aumont et al., 2005). Detailed descriptions of mechanism generation in GECKO-A can be found in Aumont et al. (2005) and Camredon et al. (2007). In this work, the GECKO-A-generated reaction mechanism for β-myrcene at 643 K demonstrated that MVK (a 1st generation product) and MACR (a 2nd generation product) formed via OH and NO3 pathways.

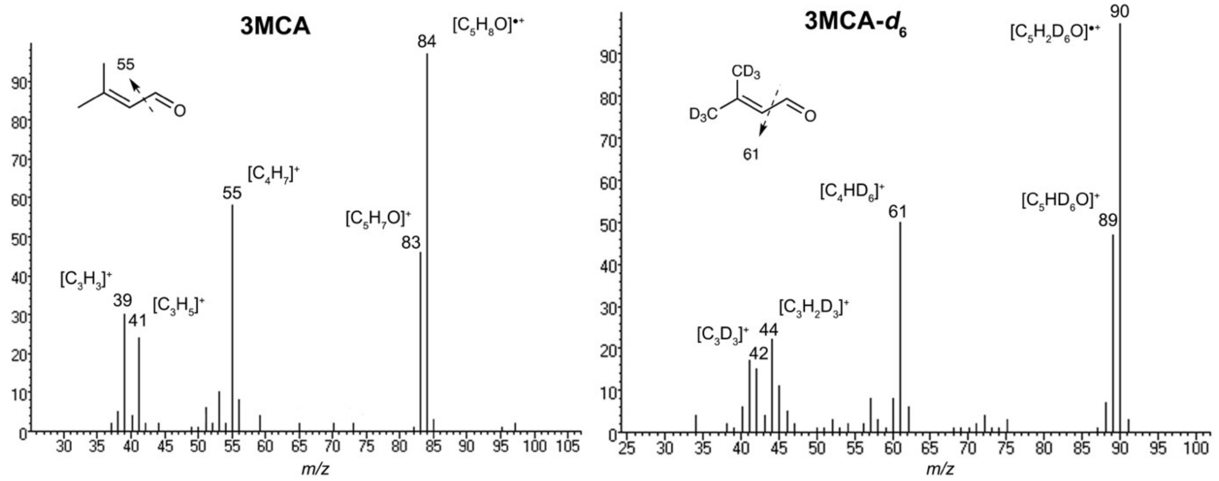

Figure S3. EIMS spectra for 3-methylacrolein (3MCA) and its deuterium isotopologue 4,4,4-trideutero-3-(1,1,1-trideuteromethyl)-prop-2-enal (3MCA-d_6) that are formed when β-myrcene-d_6 is subjected to dabbing. 3MCA-d_6 elutes immediately before 3MCA on the GC-MS chromatogram, and the structure was proposed primarily on the observation of a +6 amu mass shift on the molecular ion and a +6 amu mass shift on the isobutenyl cation.

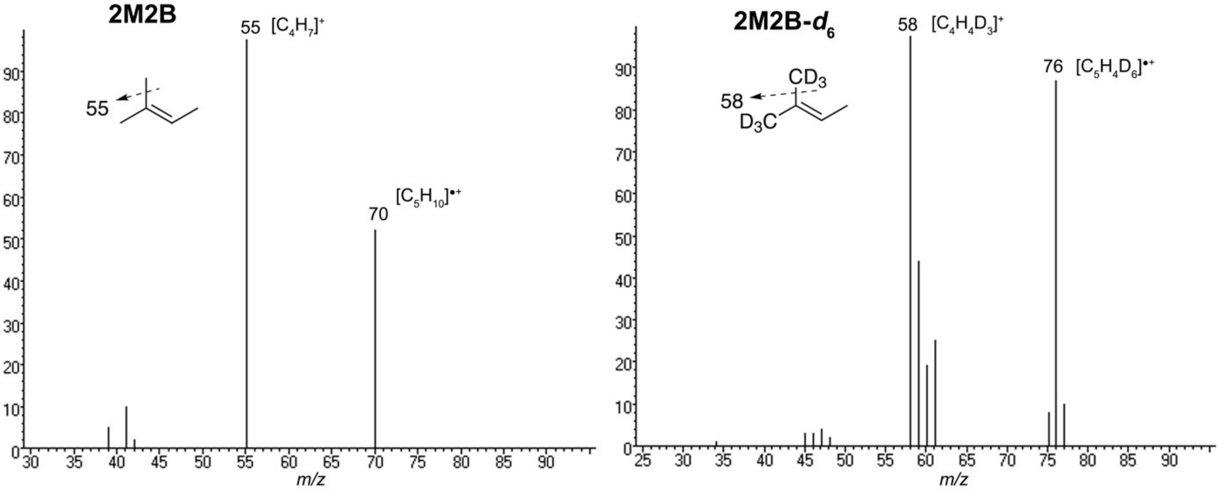

Figure S4. The EIMS spectra for 2-methyl-2-butene (2M2B) and its deuterium isotopologue 1,1,1-trideutero-2-(1,1,1-trideuteromethyl)-but-2-ene (2M2B-d_6) that are formed when β-myrcene-d_6 is subjected to dabbing. 2M2B-d_6 elutes immediately before 2M2B on the GC-MS chromatogram, and the structure was proposed primarily on the observation of a +6 amu mass shift on the molecular ion and a +3 amu mass shift on its base peak.

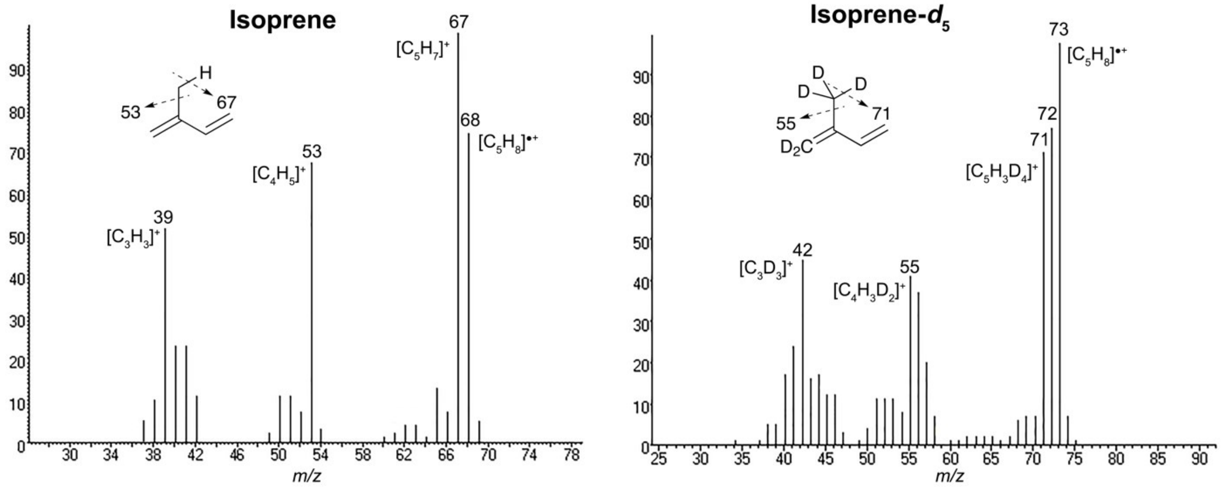

Figure S5. The EIMS spectra for isoprene and 1,1-dideutero-2-(1,1,1-trideuteromethyl)-1,3-butadiene (isoprene-d_5) that are formed when β-myrcene-d_6 is subjected to dabbing. Isoprene-d_5 elutes immediately before isoprene on the GC-MS chromatogram, and the structure was proposed primarily on the observation of a +6 amu mass shift on the molecular ion and a +2 amu mass shift on the butadienyl cation. The presence of other ions such as *m/z* = 72, 56, and 57 suggest that another isoprene-d_5 isotopomer may be present, but the relatively higher abundance of *m/z* = 73, 71, 55, and 42 suggest that the proposed structure is the most abundant isotopomer.

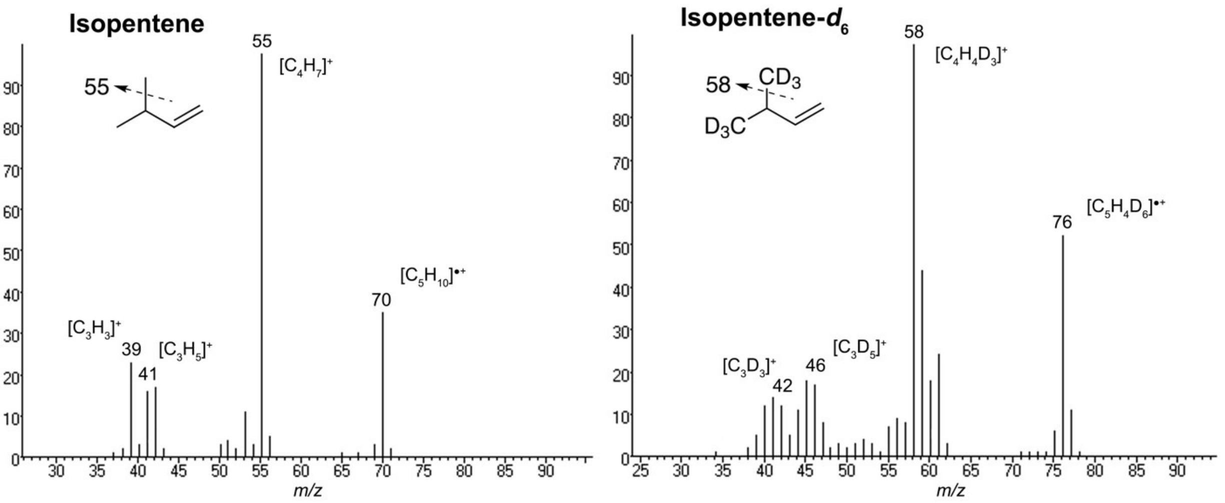

Figure S6. The EIMS spectra for isopentene and its deuterium isotopologue 4,4,4-trideutero-3-(1,1,1-trideuteromethyl)-but-1-ene (isopentene-d_6) that are formed when β-myrcene-d_6 is subjected to dabbing. Isopentene-d_6 elutes immediately before isopentene on the GC-MS chromatogram, and the structure was proposed primarily on the observation of a +6 amu mass shift on the molecular ion and a +3 amu mass shift on its base peak.

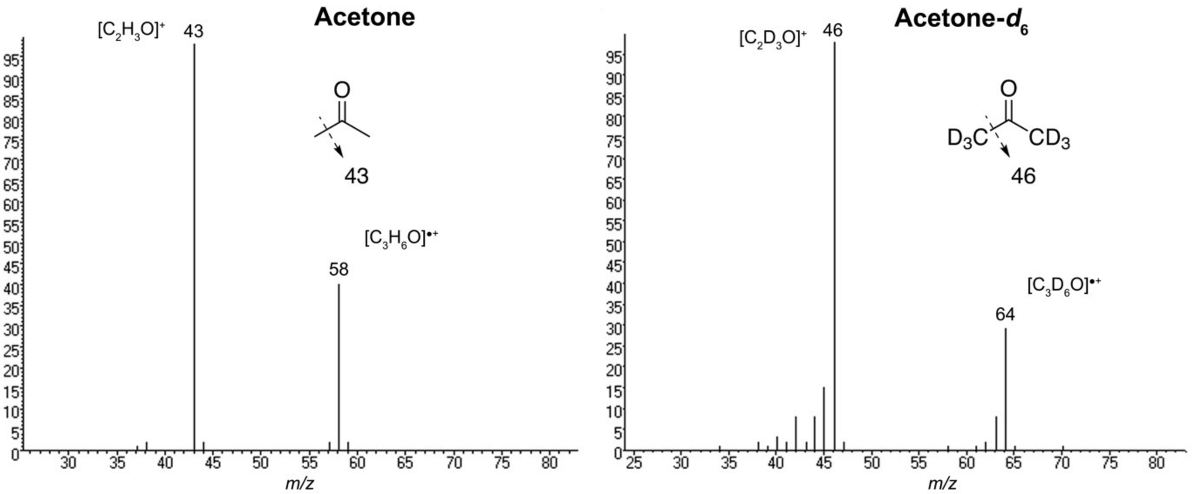

Figure S7. The EIMS spectra for acetone and its deuterium isotopologue 1,1,1,3,3,3-hexadeutero-2-propanone (acetone-d_6) that are formed when *β*-myrcene-d_6 is subjected to dabbing. Acetone-d_6 elutes immediately before acetone on the GC-MS chromatogram, and the structure was proposed primarily on the observation of a +6 amu mass shift on the molecular ion and a +3 amu mass shift on its base peak.

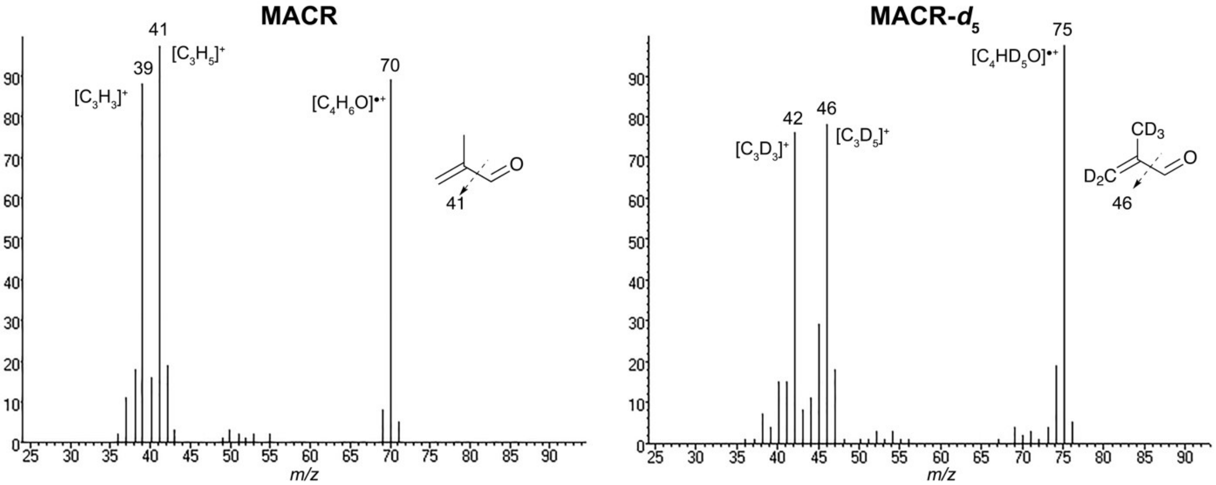

Figure S8. The EIMS spectra for methacrolein (MACR) and its deuterium isotopologue 3,3-dideutero-2-(1,1,1-trideuteromethyl)-prop-2-enal (MACR-d_5) that are formed when *β*-myrcene-d_6 is subjected to dabbing. MACR-d_5 elutes immediately before MACR on the GC-MS chromatogram, and the structure was proposed primarily on the observation of a +5 amu mass shift on the molecular ion and a +5 amu mass shift on its base peak.

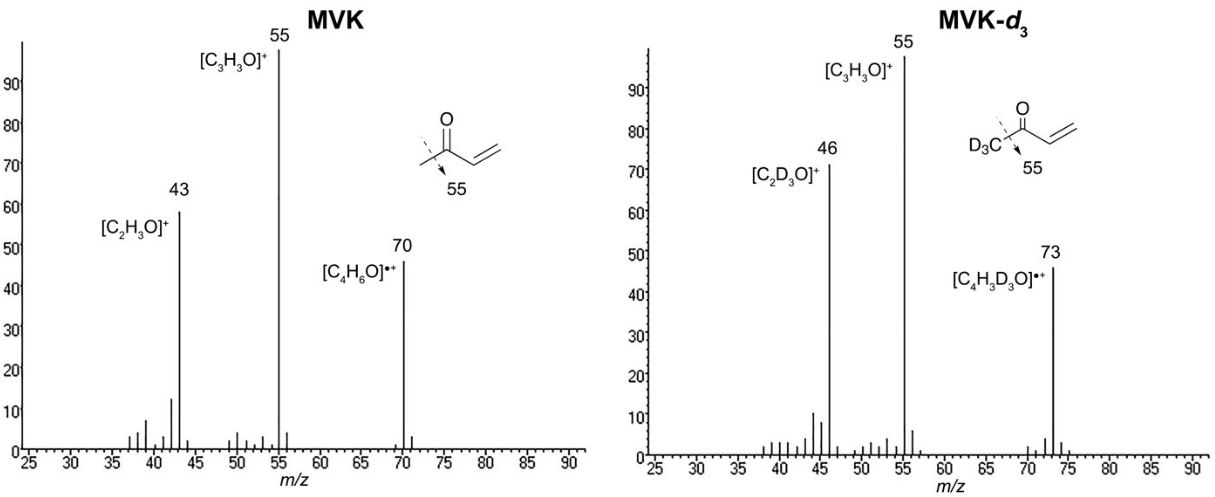

Figure S9. The EIMS spectra for methyl vinyl ketone (MVK) and its deuterium isotopologue 1,1,1-trideuterobut-3-en-2-one (MVK-d_3) that are formed when β-myrcene-d_6 is subjected to dabbing. MVK-d_3 elutes immediately before MVK on the GC-MS chromatogram, and the structure was proposed primarily on the observation of a +3 amu mass shift on the molecular ion, an identical base peak which results from loss of the methyl group, and a +3 amu mass shift on the acetyl radical.

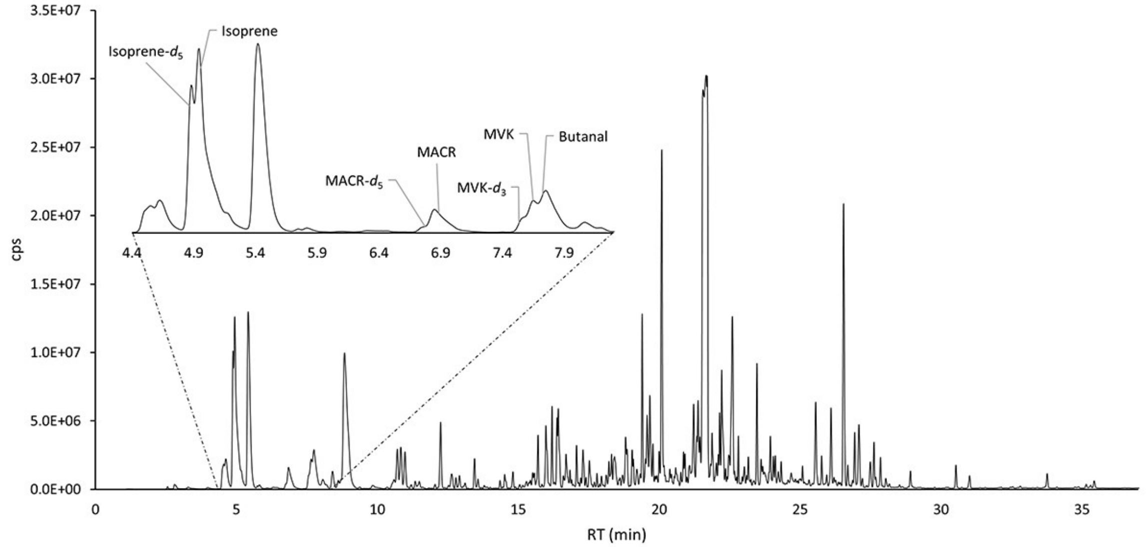

Figure S10. ATD-GC-MS chromatogram obtained from dabbing β-myrcene-d_6. The inlay highlights the presence of D-isotopologues identifiable in the chromatogram by examination of their mass spectra.

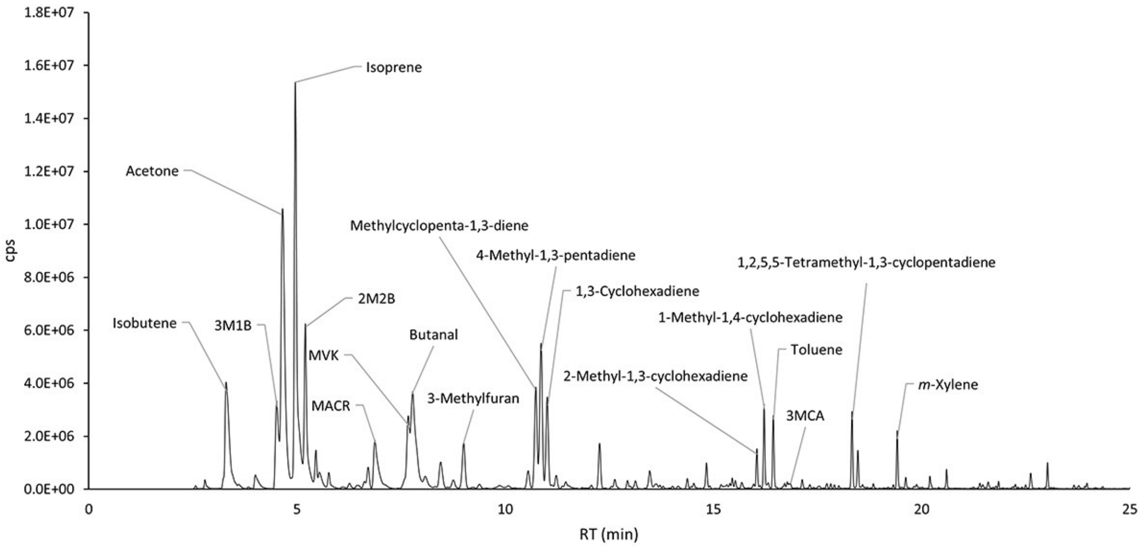

Figure S11. ATD-GC-MS chromatogram obtained from vaping pure THC.

Retention time (min)	Name	CAS #	Match quality (%)	ng analyte
**3.021**	methylethene	000115-07-1	90	2
**3.586**	isobutene	000115-11-7	90	54
**4.171**	ethanol	000064-17-5	72	3
**4.351**	1,2-dimethylcyclopropane	002402-06-4	91	37
**4.743**	(3Z)-1,3-pentadiene	001574-41-0	96	2
**5.084**	acetone	000627-20-3	55	128
**5.444**	isoprene	000078-79-5	96	1296
**5.546**	4-methyl-2-pentene	000691-38-3	87	82
**5.713**	2-methyl-2-butene	000513-35-9	91	198
**6.343**	1,4-pentadiene	000591-93-5	97	64
**7.32**	2,3-dimethyl-2-butene	000563-79-1	81	62
**7.538**	methacrolein	000078-85-3	91	34
**8.348**	2-methyl-1-pentene	000763-29-1	90	8
**8.425**	methyl vinyl ketone	000078-94-4	90	31
**8.541**	butanal	000123-72-8	94	10
**9.081**	3-vinyl-1-cyclobutene	006555-52-8	95	3
**9.351**	4-methyl-2-pentene	000674-76-0	91	357
**9.646**	(E)-3-methyl-2-pentene	000616-12-6	93	55
**9.961**	4-methyl-1,3-pentadiene	000926-56-7	95	94
**10.231**	(2Z)-3-methyl-2-pentene	000922-62-3	95	38
**10.366**	(1-methylethylidene)cyclopropane	004741-86-0	91	5
**10.951**	3,3-dimethyl-1-pentene	003404-73-7	91	17
**11.4**	4-methyl-1,3-pentadiene	000926-56-7	95	51
**11.574**	1-methyl-1,3-cyclopentadiene	000096-39-9	76	216
**11.67**	4-methyl-1,3-pentadiene	000926-56-7	95	346
**11.818**	1,4-cyclohexadiene		94	0
**12.037**	1-methyl-1-cyclopentene	000693-89-0	76	18
**12.229**	4-methylpenta-1,3-diene	000926-56-7	93	10
**12.319**	(3E)-3-methyl-3-hexene	003404-65-7	93	3
**12.39**	2,3-dimethyl-1-pentene	003404-72-6	95	12
**12.486**	hexahydrobenzene	000110-82-7	95	53
**12.808**	benzene	000071-43-2	95	12
**13.007**	1,3-cyclohexadiene	000592-57-4	87	55
**13.617**	isoprene epoxide	000000-00-0	78	6
**13.701**	(2E)-5-methyl-2-hexene	003404-62-4	74	2
**13.81**	1-heptene	000592-76-7	80	7
**14.029**	(Z)-3-methyl-3-hexene	004914-89-0	95	6
**14.119**	pentanal	000110-62-3	72	6
**14.305**	2-methyl-2-hexene	002738-19-4	91	96
**14.639**	(E)-4-methyl-2-hexene	003683-22-5	83	14
**14.819**	1,5-dimethylcyclopentene	016491-15-9	70	4

**15.012**	3-methylcyclohexene	000591-48-0	81	19
**15.481**	2-methyl-1,3-cyclohexadiene	001489-57-2	94	38
**15.821**	3-methylcyclohexene	000591-48-0	91	12
**16.008**	2,5-dihydrotoluene	004313-57-9	94	51
**16.297**	2,5-dihydrotoluene	004313-57-9	94	39
**16.438**	2-methyl-1,3,5-hexatriene	019264-50-7	95	12
**16.663**	2,5-dihydrotoluene	004313-57-9	94	36
**16.74**	1,5-dimethyl-1,4-cyclohexadiene	004190-06-1	74	7
**16.83**	1-methyl-1,4-cyclohexadiene	004313-57-9	94	81
**17.036**	toluene	000108-88-3	95	141
**17.12**	2-methyl-1,3-cyclohexadiene	001489-57-2	97	9
**17.209**	tetramethylmethylene-cyclopropane	054376-39-5	83	4
**17.287**	(3E,5E)-1,3,5-heptatriene	017679-93-5	90	22
**17.389**	6-methyl-1,5-heptadiene	007270-50-0	76	58
**17.479**	2-hexanone	000591-78-6	91	18
**17.711**	2-methyl-2-heptene	000627-97-4	95	24
**17.916**	(3E)-3-methyl-1,3,5-hexatriene	024587-26-6	94	15
**18.09**	dimethylsiloxane cyclic trimer	000541-05-9	97	30
**18.315**	(E,E,E)-2,4,6-octatriene	015192-80-0	94	31
**18.912**	5-tert-butyl-1,3-cyclopentadiene	035059-40-6	94	92
**19.054**	5-tert-butyl-1,3-cyclopentadiene	035059-40-6	91	53
**19.15**	1,2-dimethyl-1,4-cyclohexadiene	017351-28-9	87	10
**19.279**	1,4-dimethylenecyclohexane	004982-20-1	91	6
**19.426**	2,3-dimethyl-1,3-cyclohexadiene	004430-91-5	91	22
**19.574**	octa-2,4,6-triene	999178-75-1	95	7
**19.876**	2,6-dimethyl-1,5-heptadiene	006709-39-3	91	92
**19.979**	xylene	000106-42-3	97	333
**20.198**	1-methylene-3-(1-methylethylidene)cyclopentane	073913-74-3	93	31
**20.243**	1,2-dimethylenecyclohexane	002819-48-9	90	18
**20.442**	3,3,6-trimethyl-1,5-heptadiene	035387-63-4	80	173
**20.59**	o-xylene	000095-47-6	87	19
**20.699**	3-methylene-1-vinyl-1-cyclopentene	061142-07-2	76	8
**21.187**	2,3,6-trimethyl-1,5-heptadiene	033501-88-1	74	67
**21.399**	2,4-dimethyl-2,3-heptadien-5-yne	041898-89-9	81	4
**21.457**	4-methyl-1-heptene	013151-05-8	78	22
**21.56**	1-ethylnyl-2,2,3,3-tetramethylcyclopropane	103304-20-7	72	21
**21.926**	2,4-dimethyl-2,3-heptadien-5-yne	041898-89-9	91	9
**22.023**	1,4-methylethylbenzene	000622-96-8	91	8
**22.151**	2,7-dimethyl-1,6-octadiene	040195-09-3	91	264
**22.196**	beta-myrcene	000123-35-3	93	24
**22.325**	2,3,6-trimethyl-1,5-heptadiene	033501-88-1	90	65
**22.402**	1,2,5,5-tetramethyl-1,3-cyclopentadiene	004249-12-1	90	84
**22.762**	2,4-dimethyl-2,3-heptadien-5-yne	041898-89-9	70	27
**22.845**	allylbenzene	999243-49-8	86	16
**22.89**	1,2,4-trimethylenecyclohexane	014296-81-2	93	7
**23.019**	alpha-terpinolen	000586-62-9	76	6
**23.102**	p-cymene	000099-87-6	97	24
**23.231**	m-cymene	000535-77-3	93	55
**23.327**	ocimene	000502-99-8	96	12
**23.391**	eucalyptol	000470-82-6	93	6
**23.584**	m-ethyltoluene	000620-14-4	83	11
**23.648**	(3E,5E)-2,6-dimethyl-1,3,5,7-octatetraene	000460-01-5	95	5
**24.426**	4-methylbenzaldehyde	000104-87-0	94	12
**24.644**	alpha-4-dimethylstyrene	001195-32-0	98	13
**25.043**	1,3,8-para-menthatriene	018368-95-1	94	8

Table S1. All GP products from vaping THC with a CEC tentatively identified by GCMS presenting a match quality of >70 % with the NIST/Wiley mass spectral library.

**Retention time (min)**	**Name**	**CAS #**	**Match quality (%)**	**ng analyte**
**2.872**	propene	000115-07-1	86	3
**3.297**	isobutylene	000115-11-7	90	184
**3.821**	ethanol	000064-17-5	83	5

**4.001**	isopentene	001630-94-0	90	34
**4.525**	isopentene	000627-20-3	87	175
**4.654**	acetone	000067-64-1	72	661
**4.963**	isoprene	000591-95-7	95	857
**5.457**	(3Z)-1,3-pentadiene	001574-41-0	97	44
**5.766**	1,4-pentadiene	000591-93-5	97	27
**6.092**	1-propanol	000071-23-8	64	8
**6.44**	2-methylpropanal	000078-84-2	87	13
**6.629**	2,3-dimethylbut-1-ene	000563-78-0	91	7
**6.71**	2-methyl-2-pentene	000625-27-4	91	20
**6.878**	methacrolein	000078-85-3	94	238
**7.689**	methyl vinyl ketone	000078-94-4	83	224
**7.792**	butanal	000123-72-8	70	541
**8.457**	2,3-dimethylbut-2-ene	000563-79-1	76	75
**8.762**	4-methyl-1-cyclopentene	001759-81-5	91	26
**9.007**	2-methylfuran	000513-81-5	80	127
**9.38**	2,4-hexadiene	000592-46-1	94	14
**9.565**	2,3-dihydro-4-methylfuran	034314-83-5	87	4
**9.861**	tetrahydro-furan	000109-99-9	91	39
**10.552**	2,4-hexadiene	005194-51-4	94	43
**10.737**	methylcyclopenta-1,3-diene	026519-91-5	93	262
**10.865**	4-methyl-1,3-pentadiene	000926-56-7	95	339
**11.011**	1,3-cyclohexadiene	026519-91-5	93	229
**11.226**	1-methylcyclopentene	000693-89-0	93	28
**11.372**	2-butenal	004170-30-3	95	12
**11.458**	(E)-3-methyl-1,3-pentadiene	002787-43-1	90	16
**11.509**	2-butenal	004170-30-3	93	11
**11.625**	2,5-dihydrofuran	001708-29-8	80	6
**12.072**	benzene	000071-43-2	95	12
**12.269**	2-methyltetrahydrofuran	000096-47-9	60	165
**12.398**	5-methyl-1,4-hexadiene	000763-88-2	92	3
**12.552**	methyl vinyl ketone	000814-78-8	90	12
**12.939**	isoprene epoxide	000000-00-0	91	37
**13.132**	1-heptene	000592-76-7	70	31
**13.471**	pentanal	000110-62-3	91	82
**13.595**	2-(butoxymethyl)oxirane	002426-08-6	43	36
**13.716**	2,4-dimethyl-1,3-pentadiene	001000-86-8	95	8
**13.793**	(2e)-2-heptene	000592-77-8	97	6
**13.874**	oxane	000142-68-7	81	1
**13.943**	cyclopropanecarboxylic acid	001759-53-1	72	3
**14.025**	2,5-dimethylfuran	000625-86-5	93	9
**14.154**	1,5-dimethylcyclopentene	016491-15-9	70	8
**14.377**	1-methylcyclohexene	000591-49-1	87	35
**14.527**	methyl butanoate	000623-42-7	81	3
**14.591**	(Z)-cycloheptene	000628-92-2	89	4
**14.836**	1-methylcyclohexa-2,4-diene	999131-00-1	93	66
**14.913**	2,3-dimethyl-1,3-pentadiene	001113-56-0	94	5
**15.184**	1-methylcyclohexene	000591-49-1	78	13
**15.394**	1,2-dimethyl-1,3-cyclopentadiene	004784-86-5	94	15
**15.454**	(2E)-2-methyl-2-butenal	001115-11-3	91	23
**15.527**	(2E)-2-methyl-2-butenal	000497-03-0	93	29
**15.682**	(3E)-2-methyl-1,3,5-hexatriene	019264-50-7	90	26
**15.849**	(3E)-3-methyl-1,3,5-hexatriene	024587-26-6	94	5
**15.969**	2,5-dihydrotoluene	004313-57-9	83	16
**16.047**	2-methyl-1,3-cyclohexadiene	001489-57-2	94	106
**16.12**	5,6-dimethyl-1,3-cyclohexadiene	002417-81-4	91	7
**16.218**	2-methyl-1,3,5-hexatriene	019264-50-7	94	243
**16.441**	toluene	000108-88-3	95	226
**16.708**	2-methyl-1-heptene	015870-10-7	93	22
**16.776**	3-methyleneheptane	001632-16-2	94	17
**16.854**	Methylcholanthrene	000107-86-8	94	12
**17.133**	2-methyl-2-heptene	000627-97-4	91	35
**17.24**	(E)-4-octene	014850-23-8	70	3
**17.317**	2,5-dihydrotoluene	004313-57-9	93	18
**17.643**	2,5-dimethyl-1,3-hexadiene	000927-98-0	93	3
**17.725**	1,5,5-trimethyl-1,3-cyclopentadiene	999178-77-9	91	22
**17.815**	biisobutenyl	000764-13-6	92	5
**17.905**	1-methylene-2-methylcyclohexane	002808-75-5	91	13

**18.013**	3,5-dimethylcyclohexene	000823-17-6	96	11
**18.159**	(3E)-3-ethylidene-1-methyl-1-cyclopentene	062338-00-5	93	5
**18.33**	1,2,5,5-tetramethyl-1,3-cyclopentadiene	004249-12-1	91	257
**18.472**	5-tert-butyl-1,3-cyclopentadiene	035059-40-6	91	110
**18.584**	2,5-dimethylhex-5-en-3-yn-2-ol	999226-91-1	90	31
**18.841**	1,5,5-trimethyl-1,3-cyclopentadiene	999178-77-9	95	25
**18.987**	(E,E,E)-2,4,6-octatriene	015192-80-0	94	4
**19.197**	5,5-dimethyl-2-ethyl-1,3-cyclopentadiene	999221-33-9	64	6
**19.322**	2,6-dimethyl-1,5-heptadiene	006709-39-3	91	18
**19.416**	*p*-xylene	000106-42-3	97	247
**19.622**	3,3-dimethyl-6-methylenecyclohexene	020185-16-4	94	52
**19.82**	1,2-dimethyl-1,4-cyclohexadiene	017351-28-9	86	14
**19.966**	1,6-dimethylhepta-1,3,5-triene	999221-34-1	95	7
**20.017**	m-xylene	000108-38-3	60	11
**20.21**	1,5-dimethyl-1,4-cyclohexadiene	004190-06-1	90	4
**20.309**	1-methylene-3-vinylcyclohexane	999131-40-0	58	3
**20.412**	alpha-pyrone	000514-94-3	94	6
**20.893**	hexanoic acid	000142-62-1	72	6
**21.017**	1-phenylethanol	000098-85-1	76	6
**21.399**	1(7),5,8-o-menthatriene	000000-00-0	91	35
**21.46**	1,2,3-trimethylbenzene	000526-73-8	70	11
**21.545**	2,4-dimethyl-2,3-heptadien-5-yne	041898-89-9	83	6
**21.606**	2,6-dimethyl-2,7-octadiene	016736-42-8	81	24
**21.713**	3-isopropenyl-6-methyl-1-cyclohexene	005113-87-1	96	10
**21.79**	3-isopropenyl-6-methyl-1-cyclohexene	005113-87-1	98	12
**21.85**	1,6-dimethylhepta-1,3,5-triene	999221-34-1	94	37
**21.953**	octanal	000124-13-0	93	4
**22.408**	alpha-terpinene	000099-86-5	98	10
**22.498**	o-cymene	000527-84-4	97	17
**22.619**	o-cymene	000527-84-4	97	99
**22.76**	(+)-sabinene	003387-41-5	96	3
**22.82**	1,2,3-trimethylbenzene	000526-73-8	90	4
**22.962**	2,4-dimethyl-2,3-heptadien-5-yne	041898-89-9	90	7
**23.657**	3-methyl-5-methylene-norbornylene	000000-00-0	81	5
**23.846**	terpinolene	000586-62-9	96	3
**23.971**	1-methyl-2-isopropenylbenzene	001587-04-8	97	36
**24.048**	3-methylbenzaldehyde	000620-23-5	80	1
**24.147**	2-methoxy-4-methylphenol	000093-51-6	86	3
**24.353**	1,3,8-p-menthatriene	021195-59-5	93	12
**24.447**	1-methylcyclooctene	000933-11-9	94	1
**25.057**	methyl-6-methyl-8,9,10-trinorborn-5-en-2-endo-yl ketone	092356-41-7	91	17
**25.181**	methyl-6-methyl-8,9,10-trinorborn-5-en-2-endo-yl ketone	092356-41-7	91	9
**26.276**	(4-methylphenyl)ethanone	000122-00-9	94	5
**26.645**	naphthalene	000091-20-3	97	5
**26.735**	alpha-phellandren-8-ol	001686-20-0	70	2
**28.942**	2-methyl-2-norbornene	000694-92-8	83	1
**29.324**	2-methyl-2-propenoic acid	007779-31-9	72	3
**30.564**	3,4-dimethyl-7-exo-methylene-bicyclo[4.3.0]non-3-ene	999134-71-8	90	11
**31.054**	2-methylenenorbornane	000694-92-8	86	4

Table S2. All GP products from dabbing THC tentatively identified by GCMS presenting a match quality of >70 % with the NIST/Wiley mass spectral library.

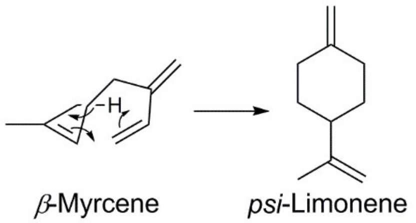

Figure S12. Proposed mechanism for the conversion of *β*-myrcene to *psi*-limonene. *psi*-Limonene formation may occur as an intramolecular ene reaction of *β*-myrcene or via a radical mechanism.

1a and 1b product distribution as a function of applied power

In order to determine the influence of applied electrical power on the product distribution of the four products deriving from radical **1** (3MCA and 2M2B from resonance structure **1a**, and isoprene and 3M1B from resonance structure **1b**), relative ratios of integrations of the molecular ion of each were graphed as a function of power.

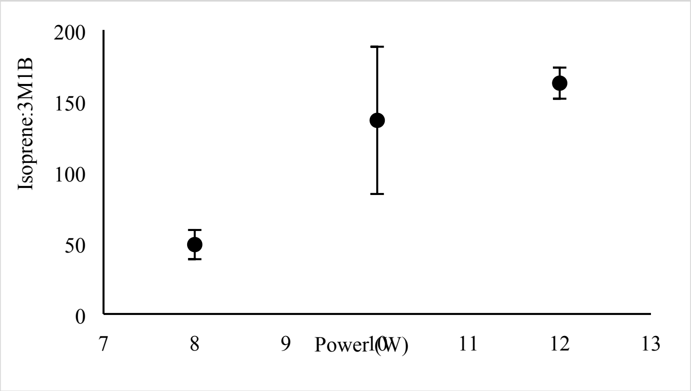

Figure S13. Relative levels of the isoprene base peak (*m/z* = 67 amu) to the 3M1B molecular ion (*m/z* = 70 amu) as a function of applied power. Note the linear increase in the isoprene:3M1B ratio with increasing power.

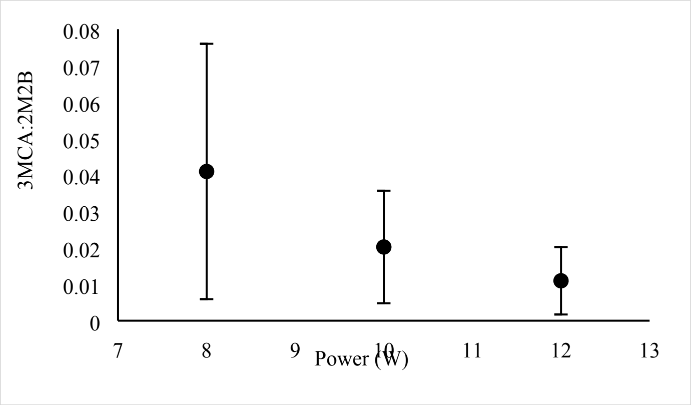

Figure S14. Relative levels of the 3MCA molecular ion (*m/z* = 84 amu) to the 2M2B molecular ion (*m/z* = 70 amu) as a function of applied power. Note the small linear decrease in the 3MCA:2M2B ratio with increasing power.

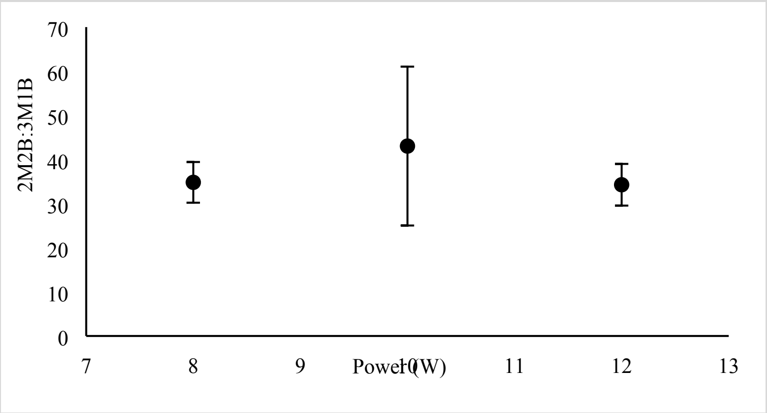

Figure S15. Relative levels of the 2M2B molecular ion (*m/z* = 70 amu) to the 3M1B molecular ion (*m/z* = 70 amu) as a function of applied power. Note this ratio does not change in a statistically significant manner with increasing power.

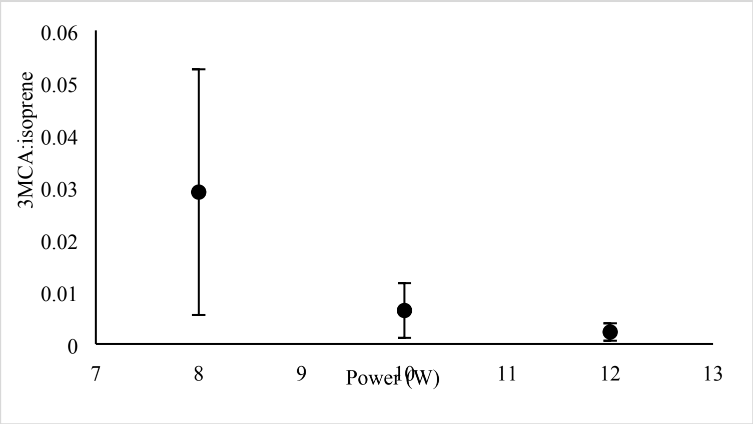

Figure S16. Relative levels of the 3MCA molecular ion (*m/z* = 84 amu) to the isoprene base peak (*m/z* = 67 amu) as a function of applied power. Note the significant decrease in the 3MCA:isoprene ratio with increasing power.

The increase in isoprene:3M1B ratio (**1b** oxidation and reduction products) with respect to power and the decrease in 3MCA:2M2B ratio (**1a** oxidation and reduction products) is mirrored by a decreasing 3MCA:isoprene ratio with respect to power. The static 2M2B:3M1B ratio signals that the decreasing **1a:1b** ratio with power is largely governed by a decreasing 3MCA:isoprene ratio.

References

1. S. M. Stevenson, R. F. Higgins, M. P. Shores and E. M. Ferreira, *Chem Sci*, 2017, **8**, 654-660.
2. K. Sun, C. Fang, W. Yang, Z. Xu, H. Wang, W. Sun, Y. Luo and Y. Xu, *J. Label. Compd. Radiopharm*, 2016, **59**, 552–556.
3. W. Liu, L. Li and C. J. Li, *Nat Comm*, 2015, **6**, 6526.
4. D. J. Comesky, D. D. Rowan and A. J. Matich, *J. Label. Compd. Radiopharm*, 2006, **49**, 47–54.
5. CORESTA, *Journal*, 2018.
6. M. Protti, V. Brighenti, M. R. Battaglia, L. Anceschi, F. Pellati and L. Mercolini, *ACS Med. Chem. Lett.*, 2019, **10**, 539-544.
7. J. Meehan-Atrash, W. Luo, K. J. McWhirter and R. M. Strongin, *ACS Omega*, 2019, **4**, 16111-16120.
8. Carter, W. P. L. The SAPRC-18 Atmospheric Chemical Mechanism. https://intra.engr.ucr.edu/~carter/SAPRC/18/ (accessed February, 2, 2020).
9. Carter, W. P. L. Documentation of the SAPRC Chemical Mechanism Modeling Software and Files. https://intra.engr.ucr.edu/~carter/SAPRC/ModelPgm.pdf (accessed February 2, 2020).
10. Carter, William P.L. (2020b). Estimates and Assignments used in the SAPRC-18 Mechanism Generation System; Report to California Air Resources Board Contract No. 11-761. In preparation. When completed, will be available at http://intra.engr.ucr.edu/~carter/SAPRC/18.
11. W. P. L. Carter, Documentation of the SAPRC Chemical Mechanism Modeling Software and Files, https://intra.engr.ucr.edu/~carter/SAPRC/ModelPgm.pdf, (accessed February 2, 2020, 2020).
12. J. Jiang, W. P. L. Carter, D. R. Cocker and K. C. Barsanti, *ACS Earth and Space Chemistry*, 2020, **4**, 1254-1268.
